# Albedo effects in the ER3BP with an oblate primary, a triaxial secondary and a potential due to belt

**DOI:** 10.1038/s41598-023-30671-3

**Published:** 2023-03-15

**Authors:** Jagadish Singh, Tyokyaa K. Richard

**Affiliations:** 1 Department of Mathematics, Faculty of Physical Sciences, Ahmadu Bello University, Zaria, Nigeria; 2 Department of Mathematical Sciences, Faculty of Physical Sciences, Federal University Dutsin-Ma, Dutsin-Ma, Katsina State Nigeria

**Keywords:** Astronomy and planetary science, Mathematics and computing, Physics

## Abstract

We have examined the effects of Albedo in the Elliptic Restricted Three-Body Problem (ER3BP) with an oblate primary, a triaxial secondary, and potential due to belt for the Earth–Moon system. We have found that as the perturbed parameters increases, the possible boundary regions of the primary come closer to one other, allowing particles to travel from one region to the next freely and possibly merge the permissible regions. Our study has revealed that the formation of triangular libration points depends on the Albedo effects, semi-major axis, the Eccentricity of the orbits, triaxiality, and the potential due to the belt. As the parameters mentioned above increase, the triangular positions ${L}_{4}$ and ${L}_{5}$ move towards the center of origin in cases 1, 2, 3, and 4 and away from the center of the origin in cases 5, 6, and 7. Considering the range of a stable and unstable libration point for the problem under study given as $0<\mu <{\mu }_{c}$ for stable libration points and ${\mu }_{c}\leq\mu \le \frac{1}{2}$ for unstable libration points, our study has established that the triangular libration points are respectively stable and unstable for cases 1, 2, and 6 and cases 3, 4, 5, and 7. Our study has also revealed that each set of values has at least one characteristic complex root with a positive real part. Hence, the triangular libration points for the Earth–Moon system are unstable in the sense of Lyapunov. The Earth–Moon system's Poincare Surface of Section (PSS) has demonstrated that a slight change in the initial conditions, the semi-major axis, and the Eccentricity of the orbits have affected the system's behavior dramatically. Further, it is seen that a chaotic dynamical behavior of the system results into either regular or irregular orbits.

## Introduction

For many years, the area of celestial mechanics has been under investigation. Newton created the three-body issue in orbital mechanics in the late seventeenth century^1,2^. In this problem, the three bodies are spherically symmetric, and they all move in response to the gravitational pull of the other two. Because of how complex the issue is, no comprehensive analytical solution exists. The idea that one of the bodies has an infinitesimal mass and the other two become the principal gravitational bodies was established by Leonhard Euler in the late eighteenth century, according to^1,2^. Therefore, the infinitesimal body will not impact the primary's motion. This transforms the issue into the well-known restricted three-body problem, greatly simplifying it.

One of the areas of celestial mechanics is the restricted three-body problem, wherein two finite bodies, known as primaries, move in elliptic or circular orbits around their centers of mass as a result of their mutual gravitational attraction, and a third body, known as infinitesimal mass, is attracted to the two primaries but is unaffected by their motion. The classical case of the restricted three-body problem has five libration points, three collinear libration points $\left({L}_{1, 2, 3}\right)$ and two non-collinear (triangular) libration points $\left({L}_{4, 5}\right)$. The collinear points are those that connect the primaries, whereas the non-collinear points are those that form equilateral triangles with them. The non-collinear libration points have been proven to be conditionally stable, whereas the collinear libration points are usually unstable^1,3–11^.

Because most celestial planets' orbits are elliptical rather than circular, the elliptic restricted three-body problem is the finest tool for analyzing the dynamical behavior of such systems. According to observations, most celestial planets are oblate spheroid or triaxial rigid bodies. The planets Earth, Jupiter, Saturn, and the stars Archid, Archerner, Anttares, Altairand, and Layten are all sufficiently oblate or triaxial rigid bodies that are important in studying celestial bodies and the Stellar System. The lack of sphericity in such celestial bodies produces a great deal of chaos.

Notable researches were carried out to ascertain the triaxiality of these heavenly bodies. Sharma and co-authors investigated stationary solutions to the planar restricted three-body problem when the primaries are triaxial rigid bodies with one of the axes serving as the axis of symmetry and the equatorial plane intersecting the plane of motion. Their findings reveal that triangular points in the same mass ratio range have long or short periodic elliptical orbits^12^. The behavior of a test particle around a triaxial primary and an oblate companion orbiting each other in elliptic orbits was studied by^13^. The primary's triaxiality and oblateness were discovered to be perturbing characteristics of the triangular points' positions and stability. The triangular points for the binaries PSR J1518 + 4904, PSR B1534 + 12, PSR B1914 + 16, and PSR B2127 + 11c are unstable due to the almost equal masses of the neutron stars. Singh and Simeon^10^ studied the triangular equilibrium points in the Circular Restricted Three-Body Problem under triaxial luminous primaries with Poynting-Robertson Drag. Duggad et al.^14^ investigated the effects of the triaxiality of both primaries on the position and stability of the oblate infinitesimal body in the elliptic restricted three-body problem. El-Bar^15^ studied the stability of collinear equilibrium points in the restricted three-body problem under some perturbations due to the bigger primary's triaxiality and the smaller primary's oblateness. Singh and Isah^16^ examined the effects of radiation pressure and triaxiality of two primaries surrounded by a belt in the Elliptic Restricted Three-Body Problem.

Broucke^17^ considered ranges of eccentricities and mass ratios as from 0 to 1 and 0 to $\frac{1}{2}$ respectively. He discovered that the periodic orbits' stability characteristics and the absence of the Jacobian integral in the elliptic problem distinguish the circular problem from the elliptic problem. Sarris^18^ investigated how families of symmetric-periodic orbits evolve and remain stable in the three-dimensional elliptic problem with changes to the mass ratio and Eccentricity of the orbits.

Peng and Xu^19^ utilized continuation methods with the multi-segment optimization method to generate two groups of multi-revolution elliptic halo (ME-Halo) orbits and then systematically investigate their stability evolution with respect to the Eccentricity and the mass ratio of the primaries.

Ferrari and Lavagna^20^ surveyed periodic solutions in the Circular Restricted Three-Body problem where both the Sun–Earth and the Earth–Moon systems were considered. Their findings supported cataloging based on the number of libration point revolutions in the periodic solutions.

Albedo is a dimensionless quantity measured on a scale from 0 to 1. A body or surface with 0 Albedo is referred to as a "black body," as it absorbs all incident radiation. A "white body" with an Albedo value of one is a perfect reflector that reflects all incident radiations entirely and uniformly in all directions. Because it reflects the bulk of the radiation that strikes it, a surface with a high Albedo has a lower temperature. Because it absorbs more incoming radiation, a surface with a low Albedo has a higher temperature. There are no black-body planets in our solar system. The planets with their respective average Albedos are shown in Table 1 below.

**Table 1:** Planets with their respective average Albedos.

Planet	Mercury	Venus	Earth	Mars	Jupiter	Saturn	Uranus	Neptune
Albedo	$0.12$	$0.75$	$0.30$	$0.16$	$0.34$	$0.34$	$0.30$	$0.29$

Sources: Idrisi and Ullah^22^.

The albedo effect is one of the fascinating non-gravitational forces that considerably impact tiny mass motion. The fraction of solar energy reflected diffusely from the planet back into space is referred to as Albedo^21^. It's a measurement of the planet's surface reflectance. The percentage of incident solar radiation reaches the planet's surface and is reflected into space according to^22–26^.

$${\text{Albedo}} = \frac{{{\text{radiation}}\;{\text{reflected}}\;{\text{back}}\;{\text{to}}\;{\text{the}}\;{\text{space}}}}{{{\text{incident}}\;{\text{radiation}}}}$$

The Earth's Albedo has an impact on several satellites. The albedo of the Earth is critical for nearly all Earth-orbiting satellites. The amount of solar radiation reflected by the Earth toward a satellite impacts the energy generated by the solar panels, which in turn affects the thermal design sensed by the horizon sensors to determine the satellite's position^27^.

In the circular restricted three-body problem, Idrisi explored how Albedo affected the libration points from their initial position^22^. When the smaller primary is a homogeneous ellipsoid, Idrisi investigated the Albedo effect on the existence and stability of the libration points^23^. The result shows that the triangular libration points are stable for $\mu <{\mu }_{c}$ where ${\mu }_{c}={\mu }_{0}-\left(0.00891747+0.222579k\right)\alpha ,$ but collinear libration points are still unstable. Idrisi and Ullah^26^ developed a model for the Elliptic restricted three-body problem in which one of the primaries is a source of radiation and the other a non-black-body. They also carried out a study to examine the significant effects of Albedo on the existence of out-of-plane equilibria in the elliptic restricted three-body problem under an oblate primary model. They found that the equilibria are unstable in a linear sense for all parameters $\mu , \alpha , e, k,$ and $\sigma$.

The belt's influence changes the dynamical system's structure, resulting in new equilibrium points under certain conditions, according to^2,15^. In the presence of a large belt, the orbital motion of a test particle around a primary is substantially influenced^16,28^. According to^12,29–31^, $\frac{{M}_{b}}{{\left({r}^{2}+{T}^{2}\right)}^\frac{1}{2}}$ is the potential due to the Belt, where ${M}_{b}$ is the total mass of the Belt, $r$ is the radial distance of the infinitesimal body, and is given by ${r}^{2}={x}^{2}+{y}^{2}, T=a+b$, where $a and b$ are parameters which determine the density profile of the Belt. The parameter $"a"$ controls the flatness of the profile and is known as the flatness parameter. The parameter $"b"$ controls the size of the core density profile and is called the core parameter. When $a=b=0$, the potential equals one by a point mass.

Singh and Taura^28^ studied the effects of oblateness up to ${J}_{4}$ in the photo-gravitational Circular Restricted Three-Body Problem with the potential due to Belt. Their results confirmed that the triangular points are stable for $0<\mu <{\mu }_{c}$ and unstable for ${\mu }_{c}\leq\mu \le \frac{1}{2}$ where $\mu$ and ${\mu }_{c}$ are respectively the mass ratio and the critical mass parameter.

In this present study, we have extended the work of^26^ to include the oblateness of the bigger primary, triaxiality of the smaller primary, and potential due to the Belt in the Earth–Moon system.

The organization of the work is in eight sections. The equations of motion are presented in “Equations of motion”; “Zero velocity curves (ZVC)” avails us with Zero Velocity Curves (ZVC), in “Location of triangular libration points”, locations of the libration points are provided; “Stability and critical mass value (μ_c_)” presents the stability and the critical mass value, Poincare Surface of Section is demonstrated in “Poincare surfaces of section (PSS)” and conclusions are drawn from “Conclusions”.

## Equations of motion

Using dimensionless variables and a barycentric synodic coordinate system $\left(\xi ,\eta , \zeta \right)$, the equations of motion of the infinitesimal mass (third body) under the effects of an oblate primary, a triaxial secondary, and a potential due to Belt can be expressed as;

1 $$\xi^{\prime\prime} - 2\eta^{\prime} = \frac{\partial \Omega }{{\partial \xi }},\eta^{\prime\prime} + 2\xi^{\prime} = \frac{\partial \Omega }{{\partial \eta }},\zeta^{\prime\prime} = \frac{\partial \Omega }{{\partial \zeta }}.$$

with the force function

2 $$\Omega ={\left(1-{e}^{2}\right)}^{-\frac{1}{2}}\left[\frac{1}{2}\left({\xi }^{2}+{\eta }^{2}\right)+\frac{1}{{n}^{2}}\left\{\frac{\left(1-\mu \right)\left(1-\alpha \right)}{{r}_{1}}+\frac{\left(1-\mu \right)\left(1-\alpha \right)A}{2{{r}_{1}}^{3}}-\frac{3\left(1-\mu \right)\left(1-\alpha \right)A{\zeta }^{2}}{2{{r}_{1}}^{5}}+\frac{\mu \left(1-\beta \right)}{{r}_{2}}+\frac{\mu \left(2{\sigma }_{1}-{\sigma }_{2}\right)\left(1-\beta \right)}{2{{r}_{2}}^{3}}-\frac{3\mu \left({\sigma }_{1}-{\sigma }_{2}\right)\left(1-\beta \right){\eta }^{2}}{2{{r}_{2}}^{5}}-\frac{3\mu \left(1-\beta \right){\sigma }_{1}{\zeta }^{2}}{2{{r}_{2}}^{5}}+\frac{{M}_{b}}{{\left({r}^{2}+{T}^{2}\right)}^\frac{1}{2}}\right\}\right].$$

3 $${{r}_{1}}^{2}={\left(\xi -{\xi }_{1}\right)}^{2}+{\eta }^{2}+{\zeta }^{2},{{r}_{2}}^{2}={\left(\xi -{\xi }_{2}\right)}^{2}+{\eta }^{2}+{\zeta }^{2}$$

where $${\xi }_{1}=\mu , { \xi }_{2}=\mu -1, 0<\mu =\frac{{M}_{2}}{{M}_{1}+{M}_{2}}<\frac{1}{2}$$.

The mean motion $"n"$ is given as

4 $${n}^{2}=\frac{{\left(1+{e}^{2}\right)}^\frac{1}{2}}{a\left(1-{e}^{2}\right)}\left[1+\frac{3A}{2}+\frac{3\left(2{\sigma }_{1}-{\sigma }_{2}\right)}{2}+\frac{2{M}_{b}{r}_{c}}{{\left({{r}_{c}}^{2}+{T}^{2}\right)}^\frac{3}{2}}\right].$$

**Figure 1:**
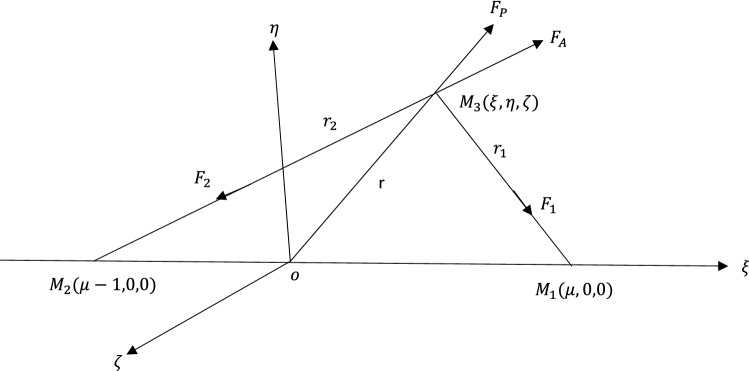
Configuration of the model.

From Fig. 1 above, ${M}_{1}$ and ${M}_{2}$ are the masses of the primaries, ${M}_{1}>{M}_{2}$. Then ${M}_{3}$ is the infinitesimal mass ${M}_{3}<<{M}_{2}$. We have that ${r}_{1}, {r}_{2}$ and $r$ are, respectively, the distances from ${M}_{1}, {M}_{2}$ and 0 to ${M}_{3}$. More so, ${F}_{1}$ and ${F}_{2}$ are the gravitational forces acting on ${M}_{3}$ due to ${M}_{1}$ and ${M}_{2}$ respectively. ${F}_{P}$ is the force due to solar radiation pressure by ${M}_{1}$ on ${M}_{3}$ and ${F}_{A}$ is the Albedo force due to solar radiation reflected by ${M}_{2}$ on ${M}_{3}$.

Now, the force acting on ${M}_{3}$ due to ${M}_{1}$ and ${M}_{2}$ are ${F}_{1}\left(1-\frac{{F}_{P}}{{F}_{1}}\right)={F}_{1}\left(1-\alpha \right)$ and ${F}_{2}\left(1-\frac{{F}_{A}}{{F}_{2}}\right)={F}_{2}\left(1-\beta \right)$ respectively. Then $\alpha =\frac{{F}_{P}}{{F}_{1}}<<1$ and $\beta =\frac{{F}_{A}}{{F}_{2}}<<1$. Also, $\alpha$ and $\beta$ can be expressed as; $\alpha =\frac{{l}_{1}}{2\pi G{M}_{1}C\sigma }; \beta =\frac{{l}_{2}}{2\pi G{M}_{2}C\sigma }$ where ${l}_{1}$ is the luminosity of the bigger primary ${M}_{1}$, ${l}_{2}$ is the luminosity of the smaller primary ${M}_{2}$, $G$ is the gravitational constant, $c$ is the speed of light, and $\sigma$ is mass per unit area of the infinitesimal mass ${M}_{3}$. Now, $\frac{\beta }{\alpha }=\frac{{M}_{1}{l}_{2}}{{M}_{2}{l}_{1}} that is \beta =\alpha \left(\frac{1-\mu }{\mu }\right)k$. Assuming $k=\frac{{l}_{2}}{{l}_{1}}=constant, 0<\alpha <1, 0<\beta <\alpha$ and $0<k<1$. Moreso, $\frac{{M}_{b}}{{\left({r}^{2}+{T}^{2}\right)}^\frac{1}{2}}$, is the potential due to belt. ${M}_{b}$ is the total mass of the belt, $r$ is the radial distance of the infinitesimal body, and is given by ${r}^{2}={\xi }^{2}+{\eta }^{2}, T=a+b$, $a and b$ are parameters which determine the density profile of the belt.

The Jacobian integral is given by

5 $$F\left(\xi ,\eta , \zeta ; \dot{\xi },\dot{\eta , }\dot{\zeta }\right)={\dot{\xi }}^{2}+{\dot{\eta }}^{2}+{\dot{\zeta }}^{2}-2\Omega +C=0.$$

where $C$ is the Jacobian constant. The mass parameter $\left(\mu \right)$ and the oblateness of the bigger primary $\left(A\right)$, as in^32^ are shown in Table 2 below.

**Table 2:** Systems and their parameters.

S./no.	System	Mass ratio $\left(\mu \right)$	Oblateness $\left(A\right)$
1	Earth–Moon	$0.0121502994$	$0.0000003686$
2	Jupiter-Io	$0.0000415283$	$0.0006701421$
3	Jupiter-Europa	$0.0000250794$	$0.0002646382$
4	Jupiter-Ganymede	$0.0000807835$	$0.0001040401$
5	Jupiter-Callisto	$0.0000479677$	$0.0000337017$
6	Saturn-Mimas	$0.0000000659$	$0.0042349996$
7	Saturn-Enceladus	$0.0000001480$	$0.0025865767$
8	Saturn-Tethys	$0.0000010950$	$0.0016835857$
9	Saturn-Dione	$0.0000020390$	$0.0010308526$
10	Saturn-Rhea	$0.0000032000$	$0.0005275432$
11	Saturn-Titan	$0.0002461294$	$0.0000981153$
12	Saturn-Hyperion	$0.0000002000$	$0.0000667989$
13	Saturn-Iapetus	$0.0000039400$	$0.0000115606$
14	Saturn-Phoebe	$0.0000000520$	$0.0000008764$

Sources: Sharma and SubbaRao^32^.

## Zero velocity curves (ZVC)

In the present study, zero velocity curves (ZVC) in the $\xi \eta$-plane is demonstrated by determining numerically the Jacobian constant $C$ using initial conditions in Eq. (5). We have observed that $2\Omega -C\ge 0$. This indicates that the curves of zero velocity are particularly defined through the expression $2\Omega =C,$ which defines a boundary relation called Hill's surface. If we consider the velocity of the infinitesimal body to be zero, then the surfaces obtained in the $\xi \eta$-plane, known as the zero relative velocity surface, can be given as;

$$C=2\Omega$$, that is

6 $${\left(1-{e}^{2}\right)}^{-\frac{1}{2}}\left[\left({\xi }^{2}+{\eta }^{2}\right)+\frac{2}{{n}^{2}}\left\{\frac{\left(1-\mu \right)\left(1-\alpha \right)}{{r}_{1}}+\frac{\left(1-\mu \right)\left(1-\alpha \right)A}{2{{r}_{1}}^{3}}-\frac{3\left(1-\mu \right)\left(1-\alpha \right)A{\zeta }^{2}}{2{{r}_{1}}^{5}}+\frac{\mu \left(1-\beta \right)}{{r}_{2}}+\frac{\mu \left(2{\sigma }_{1}-{\sigma }_{2}\right)\left(1-\beta \right)}{2{{r}_{2}}^{3}}-\frac{3\mu \left({\sigma }_{1}-{\sigma }_{2}\right)\left(1-\beta \right){\eta }^{2}}{2{{r}_{2}}^{5}}-\frac{3\mu \left(1-\beta \right){\sigma }_{1}{\zeta }^{2}}{2{{r}_{2}}^{5}}+\frac{{M}_{b}}{{\left({r}^{2}+{T}^{2}\right)}^\frac{1}{2}}\right\}\right]=C.$$

Using Table 3 below and with the help of software MATHEMATICA, the ZVC for the Earth–Moon system is demonstrated from Eq. (6) for the possible dynamics at a given Jacobian constant $C$.

**Table 3:** Numerical data in a dimensionless form for the Earth–Moon system.

S./No.	System	Mass ratio $\left(\mu \right)$	Oblateness $\left(A\right)$	Eccentricity $\left(e\right)$	Semi-major axis $\left(a\right)$
1	Earth–Moon	$0.0121502994$	$0.0000003686$	$0.0549$	$0.000452709685952$

The figures below demonstrate the effects of oblateness, Eccentricity, semi-major axis, Albedo, triaxiality, and the Belt's potential for the Earth–Moon system.

**Figure 2:**
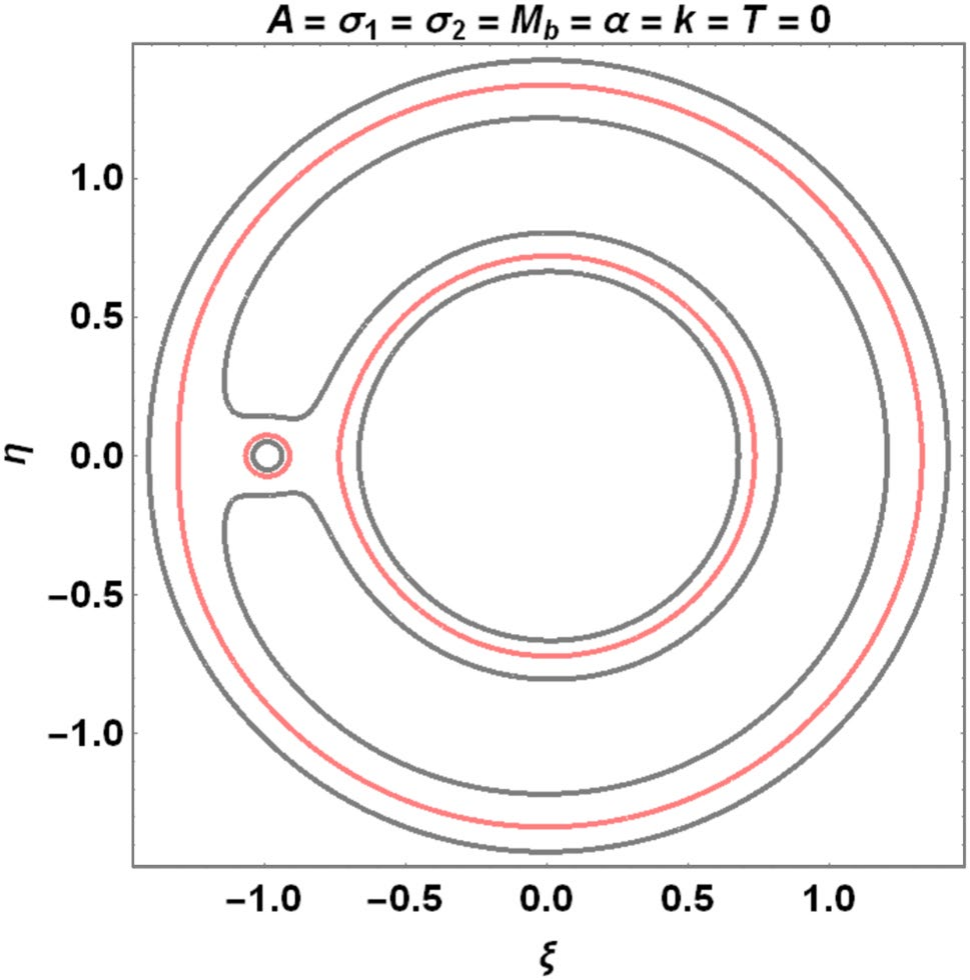
Zero velocity curves for $\mu =0.01215, e=0.0549, a=1.000453,A={\sigma }_{1}={\sigma }_{2}=\alpha =k={M}_{b}=T=0$ showing the boundary relations.

**Figure 3:**
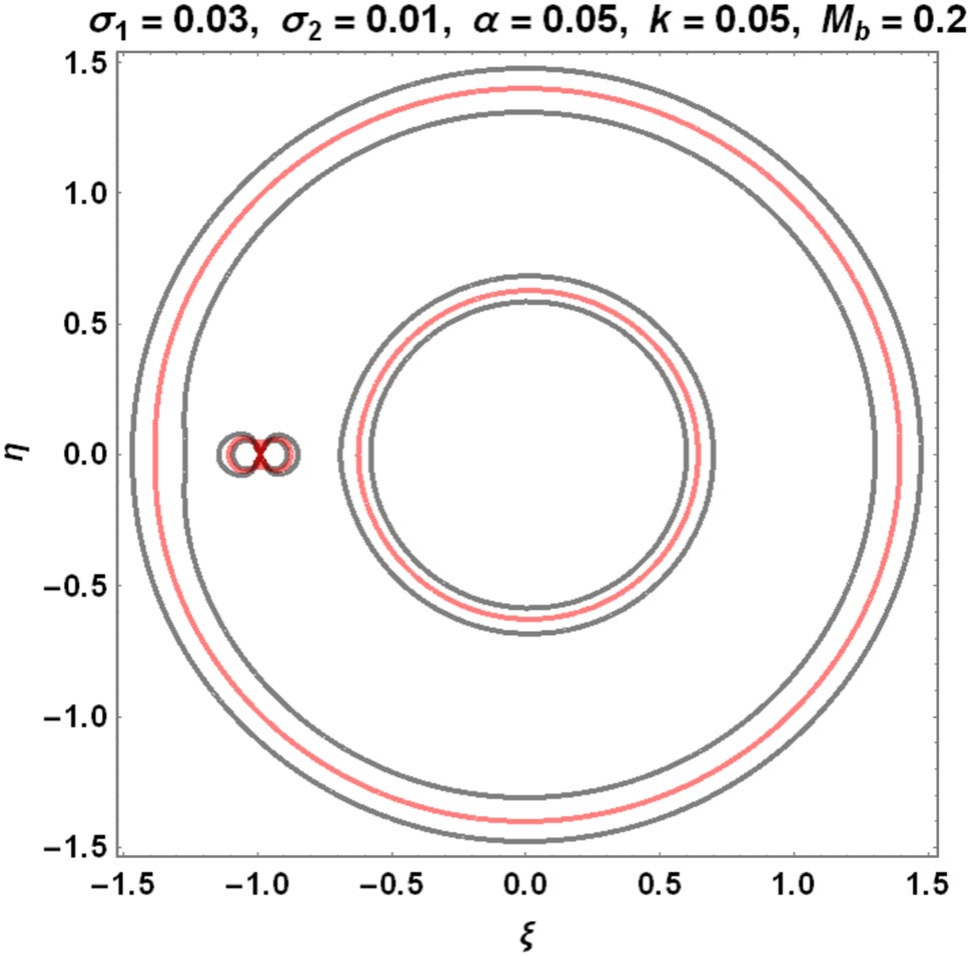
Zero velocity curves for $\mu =0.01215, e=0.0549, a=1.000453, A=0.0000003686, T=0.01, {\sigma }_{1}=0.03, {\sigma }_{2}=0.01, \alpha =0.05, k=0.05, {M}_{b}=0.2$ showing the boundary relations.

**Figure 4:**
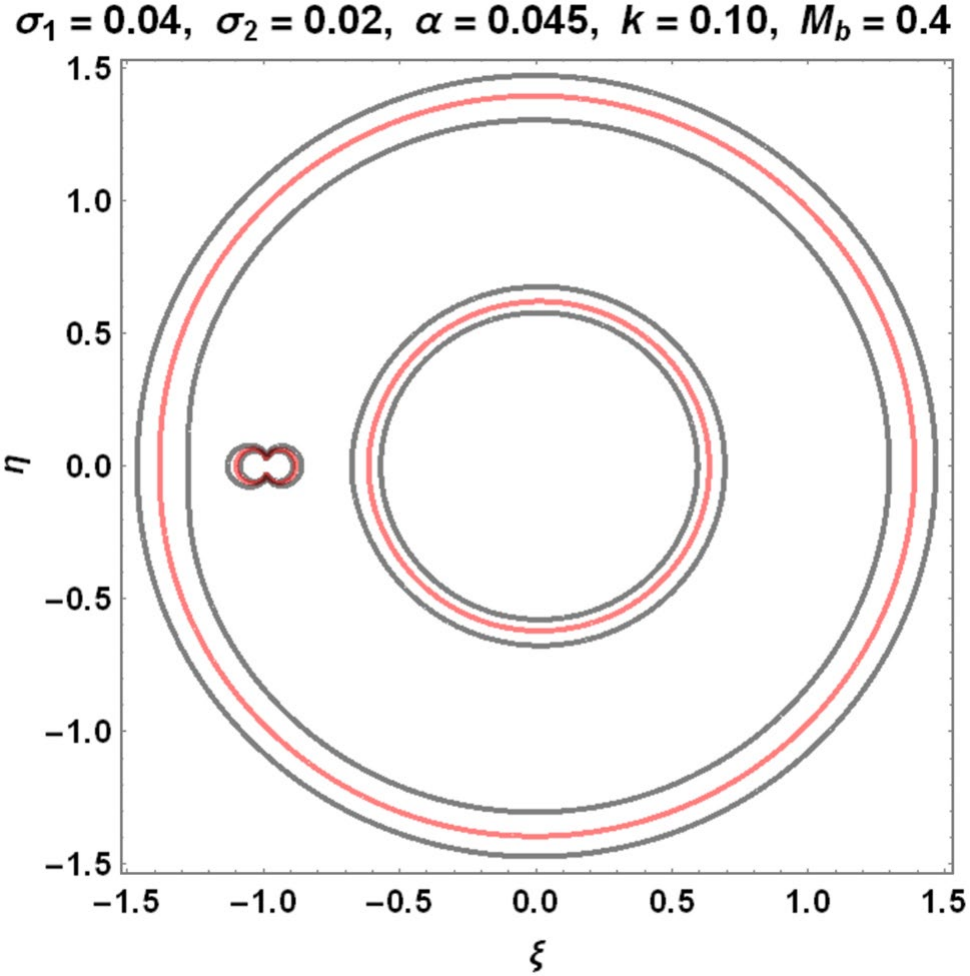
Zero velocity curves for $\mu =0.01215, e=0.0549, a=1.000453, A=0.0000003686, T=0.01, {\sigma }_{1}=0.04, {\sigma }_{2}=0.02, \alpha =0.045, k=0.10, {M}_{b}=0.4$ showing the boundary relations.

**Figure 5:**
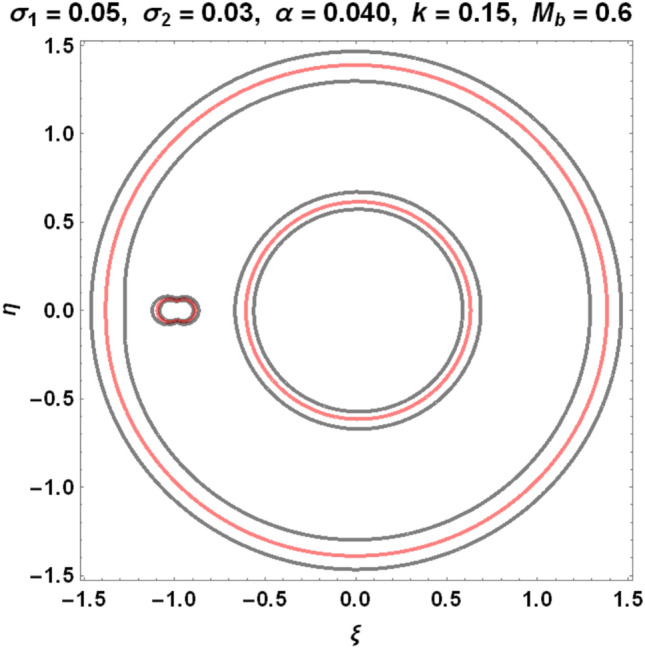
Zero velocity curves for $\mu =0.01215, e=0.0549, a=1.000453, A=0.0000003686, T=0.01, {\sigma }_{1}=0.05, {\sigma }_{2}=0.03, \alpha =0.040, k=0.15, {M}_{b}=0.6$ showing the boundary relations.

The motion of particles around the triangular libration points for the Earth–Moon system is demonstrated in Figs. 2, 3, 4 and 5. We have used Eq. (5) and Table 3 with constant and other varying values of parameters (Mass ratio, Eccentricity of the orbits, Semi-major axis, Albedo effects of both primaries, Triaxiality of the smaller primary, and the Potential due to belt as shown in Figs. 2, 3, 4 and 5) to observe the Zero velocity curves for the system under study. When the Jacobian constant C is small due to no oblateness, Albedo effects, triaxiality, and Potential due to belt, a link between the two major regions is noticed, with a small orbit forming between the openings connecting the two large regions, as shown in Fig. 2.

The single orbit created in Fig. 1 has been separated into two orbits or regions, and the two primary parts have been disconnected in the presence of the parameters mentioned above. As a result, we have witnessed a constant movement of particles within the allowed regions when the perturbed parameters are considered (see Fig. 3).

As the Albedo effects of both primaries, triaxiality of the smaller primary, and the potential due to the belt of the system increase with an increase in the Jacobian constant $C$, the two smaller obits formed in Fig. 3 merged to form a smaller region in between the two major regions resulting to three isolated possible regions (see Fig. 4).

Figure 5 shows how the movements of the major regions are affected by the values of the Jacobian constant C and other factors. The possible boundary regions of the primary get closer to each other as the Jacobian constant value increases with the perturbed parameters, allowing particles to travel freely from one zone to another and possibly merge the permissible regions.

## Location of triangular libration points

The equilibrium points are those points at which the velocity and acceleration of the particle are zero. These points are the solutions to the equations: ${\Omega }_{\xi }={\Omega }_{\eta }={\Omega }_{\zeta }=0$. This means that the solutions to equations ${\Omega }_{\xi }={\Omega }_{\eta }={\Omega }_{\zeta }=0, \eta \ne 0,\zeta =0$; are the triangular libration points. Hence, we have

7 $$\xi -\frac{1}{{n}^{2}}\left(\frac{\left(1-\mu \right)\left(1-\alpha \right)\left(\xi -\mu \right)}{{{r}_{1}}^{3}}+\frac{3\left(1-\mu \right)\left(1-\alpha \right)\left(\xi -\mu \right)A}{2{{r}_{1}}^{5}}+\frac{\mu \left(1-\beta \right)\left(\xi +1-\mu \right)}{{{r}_{2}}^{3}}+\frac{3\mu \left(2{\sigma }_{1}-{\sigma }_{2}\right)\left(1-\beta \right)\left(\xi +1-\mu \right)}{2{{r}_{2}}^{5}}-\frac{15\mu \left({\sigma }_{1}-{\sigma }_{2}\right)\left(1-\beta \right)\left(\xi +1-\mu \right){\eta }^{2}}{2{{r}_{2}}^{7}}+\frac{\xi {M}_{b}}{{\left({r}^{2}+{T}^{2}\right)}^\frac{3}{2}}\right)=0.$$

8 $$\eta \left(1-\frac{1}{{n}^{2}}\left(\frac{\left(1-\mu \right)\left(1-\alpha \right)}{{{r}_{1}}^{3}}+\frac{3\left(1-\mu \right)\left(1-\alpha \right)A}{2{{r}_{1}}^{5}}+\frac{\mu \left(1-\beta \right)}{{{r}_{2}}^{3}}+\frac{3\mu \left(2{\sigma }_{1}-{\sigma }_{2}\right)\left(1-\beta \right)}{2{{r}_{2}}^{5}}+\frac{3\mu \left({\sigma }_{1}-{\sigma }_{2}\right)\left(1-\beta \right)}{{{r}_{2}}^{5}}-\frac{15\mu \left({\sigma }_{1}-{\sigma }_{2}\right)\left(1-\beta \right){\eta }^{2}}{2{{r}_{2}}^{7}}+\frac{{M}_{b}}{{\left({r}^{2}+{T}^{2}\right)}^\frac{3}{2}}\right)\right)=0.$$

Neglecting second and higher powers of $\gamma , e, A, {\sigma }_{1}, {\sigma }_{2}$ and ${r}_{c}$ and their products, the positions of the triangular libration points ${L}_{4}(\xi , \eta )$ and ${L}_{5}\left(\xi ,- \eta \right)$ are obtained as;

9 $$\xi =\mu -\frac{1}{2}+\frac{1}{3}\left(\alpha -\beta \right)-\frac{A}{2}+\left(\frac{A}{2}\right)\alpha +\frac{1}{2}\left(\frac{-\mu }{\left(1-\mu \right)}+\frac{\mu \beta }{\left(1-\mu \right)}-\frac{3}{4}+\frac{3\beta }{4}\right){\sigma }_{1}+\frac{1}{2}\left(\frac{\mu }{\left(1-\mu \right)}-\frac{\mu \beta }{\left(1-\mu \right)}+\frac{7}{4}-\frac{7\beta }{4}\right){\sigma }_{2}.$$

where $$\beta =\frac{\alpha \left(1-\mu \right)k}{\mu }$$.

10 $$\eta =\pm \frac{\sqrt{3}}{2}\left(1-\frac{4\gamma }{9}-\frac{2}{9}\left(\alpha +\beta \right)-\frac{2{e}^{2}}{3}-\frac{A}{3}-\left(\frac{A}{3}\right)\alpha +\frac{1}{3}\left(\frac{\mu }{\left(1-\mu \right)}-\frac{\mu \beta }{\left(1-\mu \right)}-\frac{19}{4}+\frac{3\beta }{4}\right){\sigma }_{1}+\frac{1}{3}\left(\frac{-\mu }{\left(1-\mu \right)}+\frac{\mu \beta }{\left(1-\mu \right)}+\frac{15}{4}-\frac{7\beta }{4}\right){\sigma }_{2}-\frac{4{M}_{b}\left(2{r}_{c}-1\right)}{9{\left({{r}_{c}}^{2}+{T}^{2}\right)}^\frac{3}{2}}\right).$$

where $$\beta =\frac{\alpha \left(1-\mu \right)k}{\mu }$$.

**Figure 6:**
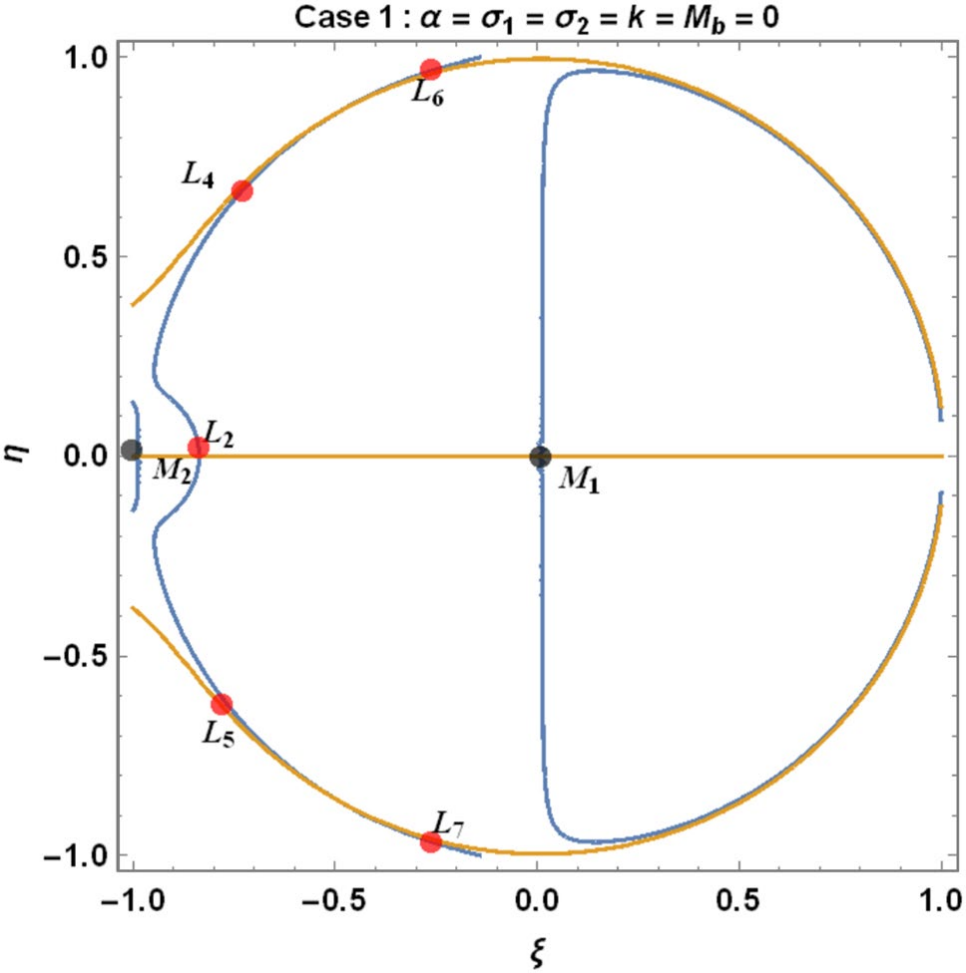
Effects of oblateness, Eccentricity of the orbits, semi-major axis, Albedo parameters, triaxiality, and the potential due to belt on the triangular libration points for the Earth–Moon system using case 1 of Table 4.

From Eqs. (9) and (10) for the Earth–Moon system, we have generated numerical values for the positions of triangular libration points using the software MATHEMATICA. The effects of the parameters involved are represented in Table 4 and demonstrated graphically in Fig. 6 below.

**Table 4:** Effects of various perturbations on the positions of libration points ${L}_{\mathrm{4,5}}\left(\mu =0.01215, A=0.0000003686, e=0.0549, \gamma =-0.0004527, T=0.01\right)$.

Case	$\alpha$	${\sigma }_{1}$	${\sigma }_{2}$	$k$	${M}_{b}$	${L}_{4}\left(\xi \right)$	${L}_{5}\left(\pm \eta \right)$
1	$0$	$0$	$0$	$0$	$0$	$-0.48785$	$0.864459$
2	$0.5$	$0$	$0$	$0$	$0$	$-0.321183$	$0.768234$
3	$0.5$	$0.03$	$0$	$0$	$0$	$-0.332618$	$0.727205$
	$0.5$	$0.04$	$0$	$0$	$0$	$-0.336429$	$0.713528$
	$0.5$	$0.05$	$0$	$0$	$0$	$-0.340241$	$0.699851$
4	$0.5$	$0.03$	$0.01$	$0$	$0$	$-0.323806$	$0.737994$
	$0.5$	$0.04$	$0.02$	$0$	$0$	$-0.318806$	$0.7351080$
	$0.5$	$0.05$	$0.03$	$0$	$0$	$-0.313806$	$0.7322210$
5	$0.5$	$0.03$	$0.01$	$0.05$	$0$	$-0.996013$	$0.3496070$
	$0.5$	$0.04$	$0.02$	$0.10$	$0$	$-1.68354$	$-0.0534018$
	$0.5$	$0.05$	$0.03$	$0.15$	$0$	$-2.39140$	$-0.468146$
6	$0.050$	$0$	$0$	$0$	$0.2$	$-0.471184$	$0.777405$
	$0.045$	$0$	$0$	$0$	$0.4$	$-0.47285$	$0.700936$
	$0.040$	$0$	$0$	$0$	$0.6$	$-0.474517$	$0.624467$
7	$0.050$	$0.03$	$0.01$	$0.05$	$0.2$	$-0.541027$	$0.708327$
	$0.045$	$0.04$	$0.02$	$0.10$	$0.4$	$-0.593300$	$0.596843$
	$0.040$	$0.05$	$0.03$	$0.15$	$0.6$	$-0.633348$	$0.492424$

**Figure 7:**
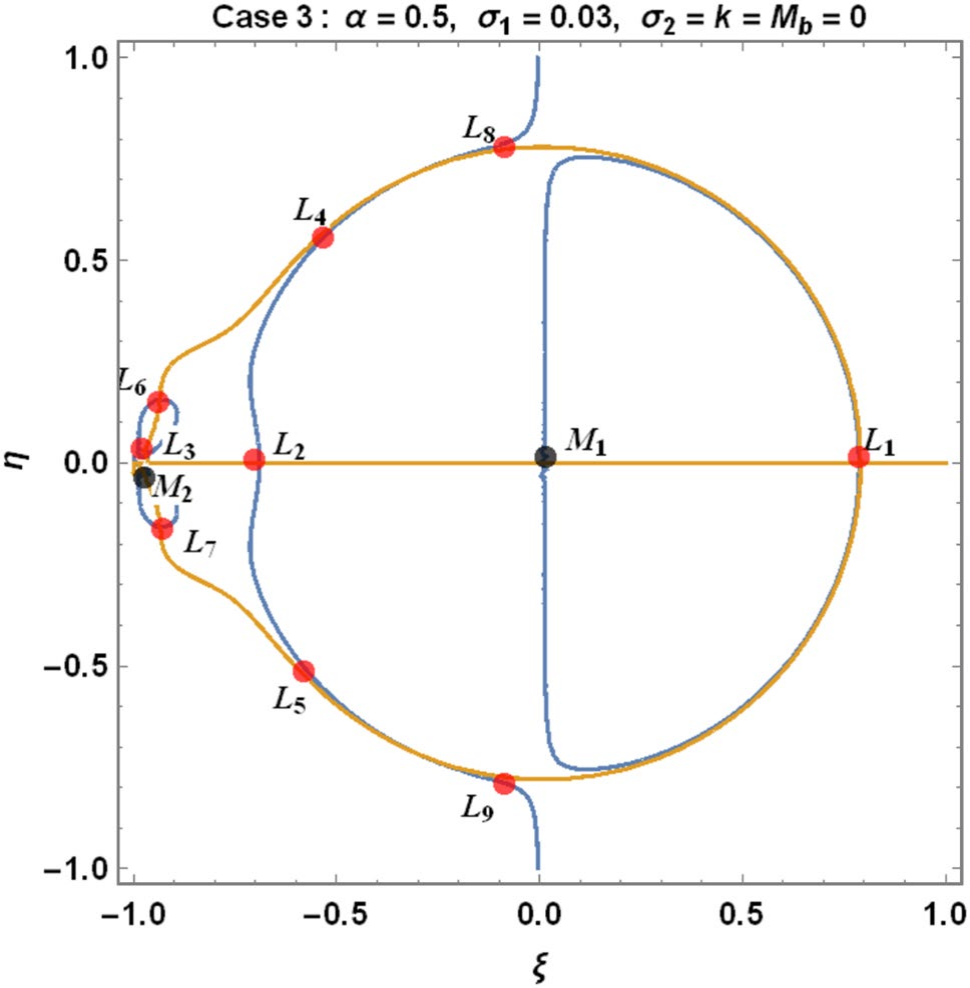
Effects of oblateness, Eccentricity of the orbits, semi-major axis, Albedo parameters, triaxiality, and the potential due to belt on the triangular libration points for the Earth–Moon system using case 3 of Table 4.

**Figure 8:**
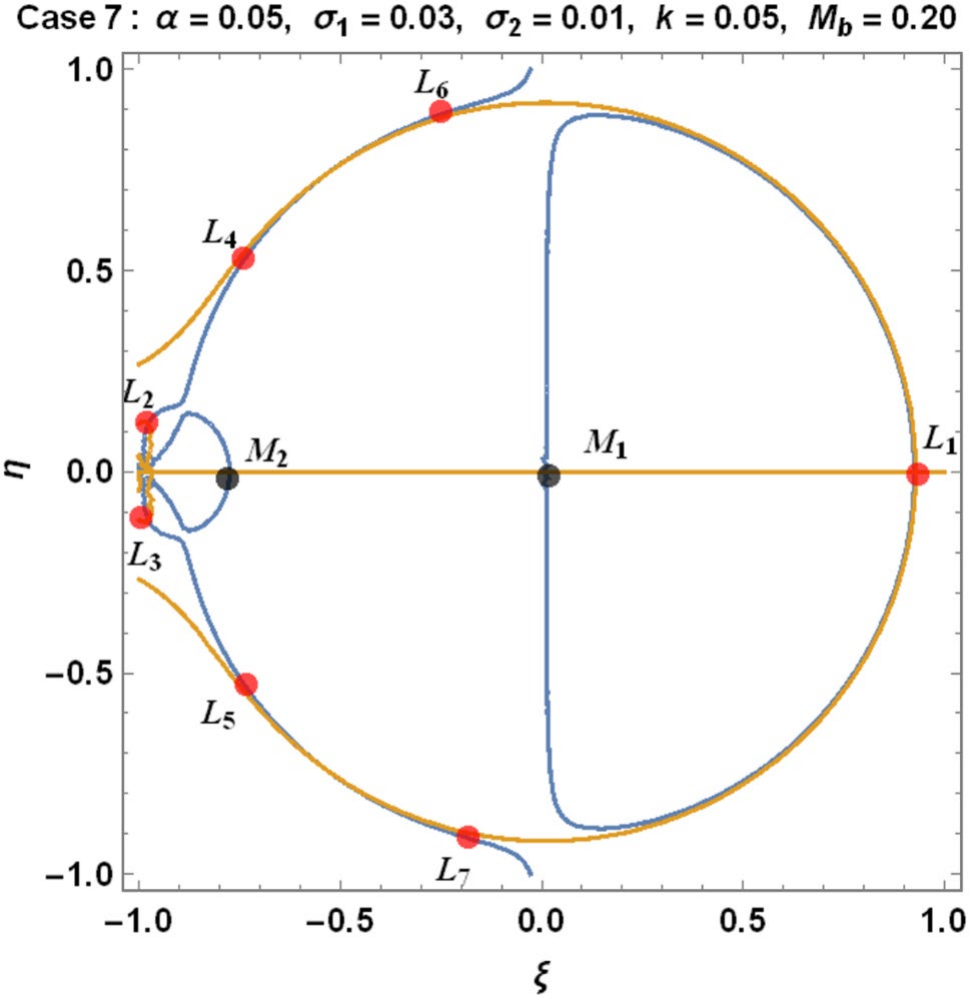
Effects of oblateness, Eccentricity of the orbits, semi-major axis, Albedo parameters, triaxiality, and the potential due to belt on the triangular libration points for the Earth–Moon system using case 7 of Table 4.

We have demonstrated the positions of libration equilibrium points when considering the solutions ${\Omega }_{\xi }={\Omega }_{\eta }=0$. The light blue line and the orange line in Figs. 6, 7 and 8, respectively, represent ${\Omega }_{\xi }=0 and {\Omega }_{\eta }=0,$ which corresponds to the equilibrium positions of the infinitesimal body ${M}_{3}$. The black dots represent the positions of the primary bodies ${M}_{i}\left(i=\mathrm{1,2},3\right)$ and the red dots are the positions of the libration points (Collinear and triangular points) denoted by ${L}_{i}\left(i=1, 2, 3, 4, 5, 6, 7, 8, 9\right)$ as in the case under study.

We have used cases 1, 3, and 7 in Table 4 to graphically demonstrate the positions of libration equilibrium points for the Earth–Moon system. In case 1, we have observed that, in the absence of Albedo effects, triaxiality of the primaries and the potential due to belt $\left(\mathrm{i}.\mathrm{e}. \alpha ={\sigma }_{1}={\sigma }_{2}=k={M}_{b}=0\right)$, the problem admits five (5) equilibrium points (see Fig. 6). Moreso, we witnessed nine (9) equilibrium points in case 3 for $\left(\alpha =0.5, {\sigma }_{1}=0.03, {\sigma }_{2}=k={M}_{b}=0\right)$ as in Fig. 7. When all the parameters $\left(\mathrm{i}.\mathrm{e}. \alpha =0.5, {\sigma }_{1}=0.03, {\sigma }_{2}=0.01, k=0.05, {M}_{b}=0.20\right)$ in our study are considered, the problem admits six (6) equilibrium points, as demonstrated in Fig. 8.

We observed from our study that the formation of these equilibrium points depends on the system parameters (Mass ratio $\left(\mu \right)$, Eccentricity $\left(e\right)$, Semi-major axis $\left(a\right)$, Oblateness $\left(A\right)$, Albedo $\left(\alpha and \beta \right)$, Triaxiality $\left({\sigma }_{1} and {\sigma }_{2}\right)$ and the Potential due to belt $\left({M}_{b}\right)$) of the problem under study.

The figure below represents the effects of oblateness, Eccentricity of the orbits, semi-major axis, Albedo parameters, triaxiality, and the potential due to belt on the triangular libration points for the Earth–Moon system. We have used seven cases of perturbations on the positions of libration points in plotting the figure below using the software MATHEMATICA. The coordinates of the triangular libration points $\left({L}_{4}, {L}_{ 5}\right)$ are: $$\begin{array}{c}\left(-0.48785, 0.864459\right),\left(-0.321183, 0.768234\right), \left(-0.332618, 0.727205\right), \left(-0.323806, 0.737994\right), \\ \left(-0.996013, 0.3496070\right), \left(-0.471184, 0.777405\right), \left(-0.541027, 0.708327\right)\end{array}$$

**Figure 9:**
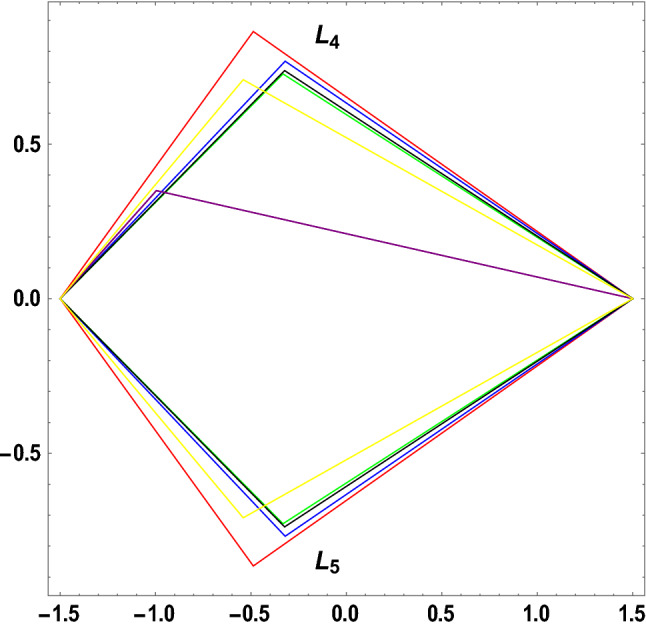
Effects of oblateness, Eccentricity of the orbits, semi-major axis, Albedo parameters, triaxiality, and the potential due to belt on the triangular libration points for the Earth–Moon system using Table 4.

Table 4 and Fig. 9 show the effects of oblateness, Eccentricity of orbits, semi-major axis, Albedo parameters, triaxiality, and the potential due to belt on the triangular libration points for the Earth–Moon system. The initial conditions $\left(\left(1.5, 0\right), \left(-1.5, 0\right)\right)$ and $\left(\left(1.5, 0\right), \left(-1.5, 0\right)\right)$ are used in plotting the graphs in Fig. 9 for the Earth–Moon system. The positions (${L}_{4} and {L}_{5}$) of the triangular libration points are affected by the aforementioned parameters for the system under study. We have observed that as the parameters increase, the position ${L}_{4}$ and ${L}_{5}$ move towards the center of origin for cases 1, 2, 3, and 4 and away from the center of origin for cases 5, 6, and 7 for the Earth–Moon system (see Table 4 and Fig. 9). As the parameters increase, constant movement occurs except in case 5, as seen in Table 4 and Fig. 9, forming different triangles.

## Stability and critical mass value (μ_c_)

The critical mass value (${\mu }_{c}$) can be obtained from the variational equations; for this, we denote the position of a dust particle near the triangular points by $\left({\xi }_{0}, {\eta }_{0}\right)$ and we let the small displacements in $\left({\xi }_{0}, {\eta }_{0}\right)$ be $\left(x,y\right)$ then $\xi ={\xi }_{0}+x$ and $\eta ={\eta }_{0}+y$, substituting these values in Eq. (1), we have variational equations

$$\ddot{x}-2\dot{y}=x{\Omega }_{\xi \xi }^{0}+y{\Omega }_{\xi \eta }^{0},$$

$$\ddot{y}+2\dot{x}=x{\Omega }_{\xi \eta }^{0}+y{\Omega }_{\eta \eta }^{0}.$$

where the subscripts denote the second partial derivatives, and the superscripts refer to the values of those derivatives at the point $\left({\xi }_{0}, {\eta }_{0}\right)$. Their characteristic equation is;

11 $${\lambda }^{4}-\left({\Omega }_{\xi \xi }^{0}+{\Omega }_{\eta \eta }^{0}-4\right){\lambda }^{2}+{\Omega }_{\xi \xi }^{0}{\Omega }_{\eta \eta }^{0}-{\left({\Omega }_{\xi \eta }^{0}\right)}^{2}=0.$$

At triangular points, we have

12 $${\Omega }_{\xi \xi }^{0}=\frac{3}{4}+\frac{\gamma }{2}+\frac{9{e}^{2}}{8}+\frac{9A}{4}-3\mu A+\left(\frac{-9\mu }{16}-\frac{3{\mu }^{2}}{2\left(1-\mu \right)}+\frac{21}{8}-\frac{75\mu \beta }{16}+\frac{{\mu }^{2}\beta }{2\left(1-\mu \right)}-\frac{9\beta }{8}\right){\sigma }_{1}+\left(\frac{87\mu }{16}+\frac{3{\mu }^{2}}{2\left(1-\mu \right)}-\frac{27}{8}+\frac{73\mu \beta }{16}-\frac{{\mu }^{2}\beta }{2\left(1-\mu \right)}+\frac{21\beta }{8}\right){\sigma }_{2}+\alpha \left(\frac{-1}{2}+\frac{3\mu }{2}-\frac{31{e}^{2}}{8}+\frac{43\mu {e}^{2}}{8}+\frac{17A}{8}+\frac{3\mu A}{8}+\left(17\mu -\frac{13}{2}\right){\sigma }_{1}+\left(\frac{-71\mu }{8}+\frac{15}{8}\right){\sigma }_{2}\right)+\beta \left(1-\frac{3\mu }{2}+\frac{3{e}^{2}}{2}-\frac{43\mu {e}^{2}}{8}+A-\frac{45\mu A}{8}+\left(2-\frac{161\mu }{16}\right){\sigma }_{1}+\left(-1+\frac{157\mu }{16}\right){\sigma }_{2}\right)-\frac{3{M}_{b}{r}_{c}}{2{\left({{r}_{c}}^{2}+{T}^{2}\right)}^\frac{3}{2}}+\frac{5{M}_{b}\left(2{r}_{c}-1\right)}{4{\left({{r}_{c}}^{2}+{T}^{2}\right)}^\frac{3}{2}}+\frac{3{M}_{b}\left(\frac{1}{4}-\mu +{\mu }^{2}\right)}{{\left({{r}_{c}}^{2}+{T}^{2}\right)}^\frac{5}{2}}.$$

13 $${\Omega }_{\eta \eta }^{0}=\frac{9}{4}-\frac{\gamma }{2}+\frac{3{e}^{2}}{8}+\frac{3A}{4}+\left(\frac{3\mu }{2\left(1-\mu \right)}+\frac{45\mu }{16}-\frac{21}{8}-\frac{3\mu \beta }{2\left(1-\mu \right)}+\frac{45\mu \beta }{32}+\frac{9\beta }{8}\right){\sigma }_{1}+\left(\frac{-3\mu }{2\left(1-\mu \right)}-\frac{45\mu }{16}+\frac{27}{8}+\frac{3\mu \beta }{2\left(1-\mu \right)}+\frac{135\mu \beta }{32}-\frac{21\beta }{8}\right){\sigma }_{2}+\alpha \left(\frac{1}{2}-\frac{3\mu }{2}-\frac{85{e}^{2}}{8}+\frac{73\mu {e}^{2}}{8}+\frac{59A}{8}-\frac{79\mu A}{8}+\left(\frac{-111}{4}+\frac{101\mu }{2}\right){\sigma }_{1}+\left(\frac{61}{4}-39\mu \right){\sigma }_{2}\right)+\beta \left(-1+\frac{3\mu }{2}-\frac{3{e}^{2}}{2}-\frac{73\mu {e}^{2}}{8}-A-\frac{71\mu A}{8}+\left(-2-\frac{861\mu }{32}+\frac{{\mu }^{2}}{\left(1-\mu \right)}\right){\sigma }_{1}+\left(1+\frac{997\mu }{32}-\frac{{\mu }^{2}}{\left(1-\mu \right)}\right){\sigma }_{2}\right)-\frac{9{M}_{b}{r}_{c}}{2{\left({{r}_{c}}^{2}+{T}^{2}\right)}^\frac{3}{2}}+\frac{7{M}_{b}\left(2{r}_{c}-1\right)}{4{\left({{r}_{c}}^{2}+{T}^{2}\right)}^\frac{3}{2}}+\frac{9{M}_{b}}{4{\left({{r}_{c}}^{2}+{T}^{2}\right)}^\frac{5}{2}}.$$

14 $${\left({\Omega }_{\xi \eta }^{0}\right)}^{2} =\frac{27}{16}-\frac{27\mu }{4}+\frac{27{\mu }^{2}}{4}+\frac{3\gamma }{4}-3\mu \gamma +3{\mu }^{2}\gamma +\frac{45{e}^{2}}{16}-\frac{45\mu {e}^{2}}{4}+\frac{45{\mu }^{2}{e}^{2}}{4}+\frac{45A}{8}-\frac{63\mu A}{4}+9{\mu }^{2}A+\left(\frac{63}{16}+\frac{9\mu }{8\left(1-\mu \right)}-\frac{369\mu }{32}+\frac{117{\mu }^{2}}{16}-\frac{9{\mu }^{2}}{8\left(1-\mu \right)}-\frac{27\beta }{16}-\frac{9\mu \beta }{8\left(1-\mu \right)}+\frac{837\mu \beta }{64}+\frac{9{\mu }^{2}\beta }{8\left(1-\mu \right)}-\frac{621{\mu }^{2}\beta }{32}-\frac{9{\mu }^{3}}{4\left(1-\mu \right)}+\frac{9{\mu }^{3}\beta }{4\left(1-\mu \right)}\right){\sigma }_{1}+\left(\frac{-81}{16}-\frac{9\mu }{8\left(1-\mu \right)}+\frac{297\mu }{32}+\frac{27{\mu }^{2}}{16}+\frac{9{\mu }^{2}}{8\left(1-\mu \right)}+\frac{63\beta }{16}+\frac{9\mu \beta }{8\left(1-\mu \right)}-\frac{1521\mu \beta }{64}-\frac{9{\mu }^{2}\beta }{8\left(1-\mu \right)}+\frac{1017{\mu }^{2}\beta }{32}+\frac{9{\mu }^{3}}{4\left(1-\mu \right)}-\frac{9{\mu }^{3}\beta }{4\left(1-\mu \right)}\right){\sigma }_{2}+\alpha \left(\frac{-3}{4}+\frac{3\mu }{4}+\frac{3{\mu }^{2}}{2}-\frac{253{e}^{2}}{16}+\frac{727\mu {e}^{2}}{16}-\frac{221{\mu }^{2}{e}^{2}}{8}+\frac{19A}{16}-\frac{355\mu A}{16}+\frac{281{\mu }^{2}A}{8}+\left(\frac{-145}{8}+\frac{821\mu }{16}-\frac{\mu }{4\left(1-\mu \right)}-\frac{2{\mu }^{2}}{\left(1-\mu \right)}-\frac{527{\mu }^{2}}{16}+\frac{3\beta }{8}+\frac{\mu \beta }{4\left(1-\mu \right)}-\frac{65\mu \beta }{32}-\frac{69{\mu }^{2}\beta }{32}+\frac{{\mu }^{2}\beta }{2\left(1-\mu \right)}+\frac{11{\mu }^{3}}{4\left(1-\mu \right)}+\frac{{\mu }^{3}\beta }{4\left(1-\mu \right)}\right){\sigma }_{1}+\left(\frac{213}{16}-\frac{558\mu }{16}+\frac{\mu }{4\left(1-\mu \right)}+\frac{2{\mu }^{2}}{\left(1-\mu \right)}+\frac{381{\mu }^{2}}{16}-\frac{7\beta }{8}-\frac{\mu \beta }{4\left(1-\mu \right)}+\frac{85\mu \beta }{32}+\frac{113{\mu }^{2}\beta }{32}-\frac{{\mu }^{2}\beta }{2\left(1-\mu \right)}-\frac{11{\mu }^{3}}{4\left(1-\mu \right)}-\frac{{\mu }^{3}\beta }{4\left(1-\mu \right)}\right){\sigma }_{2}\right)+\beta \left(\frac{3}{4}+\frac{9\mu }{4}+\frac{3{\mu }^{2}}{2}-\frac{{e}^{2}}{2}+\frac{361\mu {e}^{2}}{16}-\frac{425{\mu }^{2}{e}^{2}}{8}+\frac{5A}{2}-\frac{265\mu A}{16}+{\mu }^{2}A+\left(\frac{5}{8}+\frac{2999\mu }{64}+\frac{5\mu }{4\left(1-\mu \right)}-\frac{{\mu }^{2}}{2\left(1-\mu \right)}-\frac{2989{\mu }^{2}}{32}-\frac{3\beta }{4}-\frac{\mu \beta }{2\left(1-\mu \right)}+\frac{75\mu \beta }{16}-\frac{69{\mu }^{2}\beta }{32}-\frac{{\mu }^{2}\beta }{4\left(1-\mu \right)}-\frac{7{\mu }^{3}}{4\left(1-\mu \right)}+\frac{{\mu }^{3}\beta }{4\left(1-\mu \right)}\right){\sigma }_{1}+\left(\frac{3}{8}-\frac{4023\mu }{64}-\frac{5\mu }{4\left(1-\mu \right)}+\frac{{\mu }^{2}}{2\left(1-\mu \right)}+\frac{3741{\mu }^{2}}{32}+\frac{7\beta }{4}+\frac{\mu \beta }{2\left(1-\mu \right)}-\frac{127\mu \beta }{16}+\frac{113{\mu }^{2}\beta }{32}+\frac{{\mu }^{2}\beta }{4\left(1-\mu \right)}+\frac{7{\mu }^{3}}{4\left(1-\mu \right)}-\frac{{\mu }^{3}\beta }{4\left(1-\mu \right)}\right){\sigma }_{2}\right)-\frac{27{M}_{b}{r}_{c}}{4{\left({{r}_{c}}^{2}+{T}^{2}\right)}^\frac{3}{2}}+\frac{27\mu {M}_{b}{r}_{c}}{{\left({{r}_{c}}^{2}+{T}^{2}\right)}^\frac{3}{2}}-\frac{27{\mu }^{2}{M}_{b}{r}_{c}}{{\left({{r}_{c}}^{2}+{T}^{2}\right)}^\frac{3}{2}}+\frac{33{M}_{b}\left(2{r}_{c}-1\right)}{8{\left({{r}_{c}}^{2}+{T}^{2}\right)}^\frac{3}{2}}-\frac{33\mu {M}_{b}\left(2{r}_{c}-1\right)}{2{\left({{r}_{c}}^{2}+{T}^{2}\right)}^\frac{3}{2}}+\frac{33{\mu }^{2}{M}_{b}\left(2{r}_{c}-1\right)}{2{\left({{r}_{c}}^{2}+{T}^{2}\right)}^\frac{3}{2}}-\frac{27{M}_{b}\left(\mu -\frac{1}{2}\right)}{4{\left({{r}_{c}}^{2}+{T}^{2}\right)}^\frac{5}{2}}+\frac{27\mu {M}_{b}\left(\mu -\frac{1}{2}\right)}{2{\left({{r}_{c}}^{2}+{T}^{2}\right)}^\frac{5}{2}}.$$

Considering Eqs. (12) to (14) into Eq. (11), we have;

15 $${\lambda }^{4}+\left(4-3{\in }_{1}\right){\lambda }^{2}+\frac{27\mu }{4}\left(1-\mu \right)+{\in }_{2}=0.$$

where;

$${\in }_{1}=1+\frac{{e}^{2}}{2}+A-\mu A+\left(\frac{3\mu }{4}+\frac{\mu }{2\left(1-\mu \right)}-\frac{{\mu }^{2}}{2\left(1-\mu \right)}-\frac{35\mu \beta }{32}-\frac{\mu \beta }{2\left(1-\mu \right)}+\frac{{\mu }^{2}\beta }{6\left(1-\mu \right)}\right){\sigma }_{1}+\left(\frac{7\mu }{8}-\frac{\mu }{2\left(1-\mu \right)}+\frac{{\mu }^{2}}{2\left(1-\mu \right)}+\frac{281\mu \beta }{96}+\frac{\mu \beta }{2\left(1-\mu \right)}-\frac{{\mu }^{2}\beta }{6\left(1-\mu \right)}\right){\sigma }_{2}+\alpha \left(\frac{-29{e}^{2}}{12}+\frac{29\mu {e}^{2}}{12}+\frac{19A}{12}-\frac{19\mu A}{12}+\left(\frac{-137}{12}+\frac{45\mu }{2}\right){\sigma }_{1}+\left(\frac{137}{24}-\frac{383\mu }{24}\right){\sigma }_{2}\right)+\beta \left(\frac{-29\mu {e}^{2}}{12}-\frac{29\mu A}{12}+\left(\frac{-1183\mu }{96}+\frac{{\mu }^{2}}{3\left(1-\mu \right)}\right){\sigma }_{1}+\left(\frac{437\mu }{32}-\frac{{\mu }^{2}}{3\left(1-\mu \right)}\right){\sigma }_{2}\right)-\frac{2{M}_{b}{r}_{c}}{{\left({{r}_{c}}^{2}+{T}^{2}\right)}^\frac{3}{2}}+\frac{{M}_{b}}{{\left({{r}_{c}}^{2}+{T}^{2}\right)}^\frac{3}{2}}\left(2{r}_{c}-1\right)+\frac{{M}_{b}}{{\left({{r}_{c}}^{2}+{T}^{2}\right)}^\frac{5}{2}}-\frac{\mu {M}_{b}}{{\left({{r}_{c}}^{2}+{T}^{2}\right)}^\frac{5}{2}}+\frac{{\mu }^{2}{M}_{b}}{{\left({{r}_{c}}^{2}+{T}^{2}\right)}^\frac{5}{2}}.$$

$${\in }_{2}=3\mu \gamma -3{\mu }^{2}\gamma +\frac{45\mu {e}^{2}}{4}-\frac{45{\mu }^{2}{e}^{2}}{4}+9\mu A-9{\mu }^{2}A+\left(\frac{99\mu }{8}-\frac{117{\mu }^{2}}{16}-\frac{2889\mu \beta }{128}+\frac{621{\mu }^{2}\beta }{32}\right){\sigma }_{1}+\left(\frac{27\mu }{32}-\frac{27{\mu }^{2}}{16}+\frac{4761\mu \beta }{128}-\frac{1017{\mu }^{2}\beta }{32}\right){\sigma }_{2}+\alpha \left(\frac{3\mu }{2}-\frac{3{\mu }^{2}}{2}-\frac{{e}^{2}}{2}-\frac{221\mu {e}^{2}}{8}+\frac{221{\mu }^{2}{e}^{2}}{8}+\frac{79A}{8}+\frac{95\mu A}{8}-\frac{245{\mu }^{2}A}{8}+\left(\frac{-235}{16}+\frac{61\mu }{4}+38{\mu }^{2}-\frac{3\beta }{2}+\frac{151\mu \beta }{64}+\frac{723{\mu }^{2}\beta }{64}\right){\sigma }_{1}+\left(\frac{-33}{32}-\frac{3\mu }{32}-\frac{579{\mu }^{2}}{16}+\frac{7\beta }{2}-\frac{663\mu \beta }{64}-\frac{259{\mu }^{2}\beta }{64}\right){\sigma }_{2}\right)+\beta \left(\frac{3}{4}-\frac{45\mu }{8}-\frac{3{\mu }^{2}}{2}+\frac{31{e}^{2}}{8}-\frac{1109\mu {e}^{2}}{32}+\frac{425{\mu }^{2}{e}^{2}}{8}-\frac{A}{4}+\frac{125\mu A}{32}-{\mu }^{2}A+\left(\frac{5}{4}-\frac{251\mu }{4}+\frac{1427{\mu }^{2}}{16}+\frac{15\beta }{8}-\frac{159\mu \beta }{32}+\frac{3{\mu }^{2}\beta }{64}\right){\sigma }_{1}+\left(\frac{3}{4}+\frac{1233\mu }{16}-\frac{1803{\mu }^{2}}{16}-\frac{35\beta }{8}+\frac{515\mu \beta }{32}-\frac{631{\mu }^{2}\beta }{64}\right){\sigma }_{2}\right)-\frac{27\mu {M}_{b}{r}_{c}}{{\left({{r}_{c}}^{2}+{T}^{2}\right)}^\frac{3}{2}}+\frac{27{\mu }^{2}{M}_{b}{r}_{c}}{{\left({{r}_{c}}^{2}+{T}^{2}\right)}^\frac{3}{2}}-\frac{3{M}_{b}\left(2{r}_{c}-1\right)}{2{\left({{r}_{c}}^{2}+{T}^{2}\right)}^\frac{3}{2}}+\frac{33\mu {M}_{b}\left(2{r}_{c}-1\right)}{2{\left({{r}_{c}}^{2}+{T}^{2}\right)}^\frac{3}{2}}-\frac{33{\mu }^{2}{M}_{b}\left(2{r}_{c}-1\right)}{2{\left({{r}_{c}}^{2}+{T}^{2}\right)}^\frac{3}{2}}+\frac{27\mu {M}_{b}}{4{\left({{r}_{c}}^{2}+{T}^{2}\right)}^\frac{5}{2}}-\frac{27{\mu }^{2}{M}_{b}}{4{\left({{r}_{c}}^{2}+{T}^{2}\right)}^\frac{5}{2}}.$$

Introducing ${\lambda }^{2}=\Lambda$, then Eq. (15) becomes

16 $${\Lambda }^{2}+P\Lambda +Q=0.$$

where $$P=\left(4-3{\in }_{1}\right)$$, $$Q=\frac{27\mu }{4}\left(1-\mu \right)+{\in }_{2}$$.

To determine the value of the critical mass, we consider the discriminant of Eq. (16), that is,

17 $$\Delta ={P}^{2}-4Q.$$

Solving and neglecting the higher power of $\gamma , {e}^{2}, A, \alpha , \beta , {\sigma }_{1}, {\sigma }_{2}, {M}_{b}$ and their products, we have;

18 $$\Delta =\left[ 27+12\gamma +45{e}^{2}+36A+\frac{117{\sigma }_{1}}{4}+\frac{27{\sigma }_{2}}{4}+\alpha \left(6-\frac{221{e}^{2}}{2}+\frac{245A}{2}-152{\sigma }_{1}+\frac{579{\sigma }_{2}}{4}\right)+\beta \left(6-\frac{425{e}^{2}}{2}+4A-431{\sigma }_{1}+\frac{2181{\sigma }_{2}}{4}\right)-\frac{108{M}_{b}{r}_{c}}{{\left({{r}_{c}}^{2}+{T}^{2}\right)}^\frac{3}{2}}+\frac{66{M}_{b}}{{\left({{r}_{c}}^{2}+{T}^{2}\right)}^\frac{3}{2}}\left(2{r}_{c}-1\right)+\frac{21{M}_{b}}{{\left({{r}_{c}}^{2}+{T}^{2}\right)}^\frac{5}{2}}\right]{\mu }^{2}+\left[-27-12\gamma -45{e}^{2}-30A-54{\sigma }_{1}-\frac{69{\sigma }_{2}}{8}+\alpha \left(-6+96{e}^{2}-38A-196{\sigma }_{1}+\frac{769{\sigma }_{2}}{8}\right)+\beta \left(\frac{9}{2}+176{e}^{2}-\frac{135A}{4}+\frac{969{\sigma }_{1}}{2}-608{\sigma }_{2}\right)+\frac{108{M}_{b}{r}_{c}}{{\left({{r}_{c}}^{2}+{T}^{2}\right)}^\frac{3}{2}}-\frac{66{M}_{b}}{{\left({{r}_{c}}^{2}+{T}^{2}\right)}^\frac{3}{2}}\left(2{r}_{c}-1\right)-\frac{21{M}_{b}}{{\left({{r}_{c}}^{2}+{T}^{2}\right)}^\frac{5}{2}}\right]\mu +\left[1-3{e}^{2}-6A+\alpha \left(\frac{33{e}^{2}}{2}-49A+\frac{509{\sigma }_{1}}{4}-\frac{241{\sigma }_{2}}{8}\right)+\beta \left(-8{e}^{2}+\frac{319A}{4}+\frac{23{\sigma }_{1}}{2}-\frac{39{\sigma }_{2}}{2}\right)+\frac{12{M}_{b}{r}_{c}}{{\left({{r}_{c}}^{2}+{T}^{2}\right)}^\frac{3}{2}}-\frac{6{M}_{b}}{{\left({{r}_{c}}^{2}+{T}^{2}\right)}^\frac{3}{2}}\left(2{r}_{c}-1\right)-\frac{6{M}_{b}}{{\left({{r}_{c}}^{2}+{T}^{2}\right)}^\frac{5}{2}}\right].$$

There is only one value of $\mu$, say ${\mu }_{c}$ in $\left(0, \frac{1}{2}\right)$ for which $\Delta$ vanishes. The triangular points are stable for $0<\mu <{\mu }_{c}$ and unstable for ${\mu }_{c}<\mu \le \frac{1}{2}$ by^33^. Now, we are interested in the discriminant being zero at the critical point. Considering Eq. (17), we have $\Delta =0$ that is;

19 $$\left[27+12\gamma +45{e}^{2}+36A+\frac{117{\sigma }_{1}}{4}+\frac{27{\sigma }_{2}}{4}+\alpha \left(6-\frac{221{e}^{2}}{2}+\frac{245A}{2}-152{\sigma }_{1}+\frac{579{\sigma }_{2}}{4}\right)+\beta \left(6-\frac{425{e}^{2}}{2}+4A-431{\sigma }_{1}+\frac{2181{\sigma }_{2}}{4}\right)-\frac{108{M}_{b}{r}_{c}}{{\left({{r}_{c}}^{2}+{T}^{2}\right)}^\frac{3}{2}}+\frac{66{M}_{b}}{{\left({{r}_{c}}^{2}+{T}^{2}\right)}^\frac{3}{2}}\left(2{r}_{c}-1\right)+\frac{21{M}_{b}}{{\left({{r}_{c}}^{2}+{T}^{2}\right)}^\frac{5}{2}}\right]{\mu }^{2}+\left[-27-12\gamma -45{e}^{2}-30A-54{\sigma }_{1}-\frac{69{\sigma }_{2}}{8}+\alpha \left(-6+96{e}^{2}-38A-196{\sigma }_{1}+\frac{769{\sigma }_{2}}{8}\right)+\beta \left(\frac{9}{2}+176{e}^{2}-\frac{135A}{4}+\frac{969{\sigma }_{1}}{2}-608{\sigma }_{2}\right)+\frac{108{M}_{b}{r}_{c}}{{\left({{r}_{c}}^{2}+{T}^{2}\right)}^\frac{3}{2}}-\frac{66{M}_{b}}{{\left({{r}_{c}}^{2}+{T}^{2}\right)}^\frac{3}{2}}\left(2{r}_{c}-1\right)-\frac{21{M}_{b}}{{\left({{r}_{c}}^{2}+{T}^{2}\right)}^\frac{5}{2}}\right]\mu +\left[1-3{e}^{2}-6A+\alpha \left(\frac{33{e}^{2}}{2}-49A+\frac{509{\sigma }_{1}}{4}-\frac{241{\sigma }_{2}}{8}\right)+\beta \left(-8{e}^{2}+\frac{319A}{4}+\frac{23{\sigma }_{1}}{2}-\frac{39{\sigma }_{2}}{2}\right)+\frac{12{M}_{b}{r}_{c}}{{\left({{r}_{c}}^{2}+{T}^{2}\right)}^\frac{3}{2}}-\frac{6{M}_{b}}{{\left({{r}_{c}}^{2}+{T}^{2}\right)}^\frac{3}{2}}\left(2{r}_{c}-1\right)-\frac{6{M}_{b}}{{\left({{r}_{c}}^{2}+{T}^{2}\right)}^\frac{5}{2}}\right]=0.$$

Equation (19) is a quadratic equation in $\mu$. Solving it for $\mu$, we obtain the critical mass value ${\mu }_{c}$ as thus;

20 $${\mu }_{c}=\frac{1}{2}\left(1-\sqrt{\frac{23}{27}} \right)-\left(\frac{4}{27\sqrt{69}}\right)\gamma -\left(\frac{14}{9\sqrt{69}}\right){e}^{2}-\frac{1}{9}\left(1+\frac{13}{\sqrt{69}}\right)A+\frac{11}{24}\left(1-\frac{3553}{3\sqrt{69}}\right){\sigma }_{1}+\frac{5}{48}\left(\frac{1}{3}-\frac{19}{\sqrt{69}}\right){\sigma }_{2}+\alpha \left[-\left(\frac{2}{27\sqrt{69}}\right)-\frac{13}{108}\left(\frac{1}{3}-\frac{991}{\sqrt{69}}\right){e}^{2}-\frac{1}{324}\left(595-\frac{221}{\sqrt{69}}\right)A+\frac{1}{36}\left(\frac{659}{3}-\frac{355}{\sqrt{69}}\right){\sigma }_{1}-\frac{1}{48}\left(\frac{5863}{27}-\frac{1395}{\sqrt{69}}\right){\sigma }_{2}\right]+\beta \left[-\frac{1}{108}\left(21-\frac{181}{\sqrt{69}}\right)+\frac{1}{27}\left(\frac{56}{3}-\frac{233}{2\sqrt{69}}\right){e}^{2}+\frac{1}{648}\left(349+\frac{21307}{\sqrt{69}}\right)A-\frac{1}{24}\left(\frac{485}{18}-\frac{335}{\sqrt{69}}\right){\sigma }_{1}-\frac{1}{24}\left(\frac{533}{18}+\frac{2233}{4\sqrt{69}}\right){\sigma }_{2}\right]+\frac{16}{3\sqrt{69}}\frac{{M}_{b}{r}_{c}}{{\left({{r}_{c}}^{2}+{T}^{2}\right)}^\frac{3}{2}}-\frac{76}{27\sqrt{69}}\frac{{M}_{b}}{{\left({{r}_{c}}^{2}+{T}^{2}\right)}^\frac{3}{2}}\left(2{r}_{c}-1\right)-\frac{61}{27\sqrt{69}}\frac{{M}_{b}}{{\left({{r}_{c}}^{2}+{T}^{2}\right)}^\frac{5}{2}}.$$

The Albedo effects, triaxiality, potential due to belt, and other constant parameters on the critical mass value ${\mu }_{c}$ are demonstrated in Eq. (20).

With the help of the software MATHEMATICA, we have computed the critical mass value numerically ${\mu }_{c}$ from Eq. (20) considering the Earth–Moon system. The effects of the parameters on the ${\mu }_{c}$ are represented in Table 5. Now, considering the range of stable and unstable libration points for the problem under study given as $0<\mu <{\mu }_{c}$ for stable libration points and ${\mu }_{c}\leq\mu \le \frac{1}{2}$ for unstable libration points, our study confirmed that the triangular libration points are stable and unstable for cases 1, 2, 6, and cases 3, 4, 5, and 7, respectively (see Table 5). When some of the perturbed parameters are absent, the stability region of the triangular libration points increases, while its stability region decreases in the presence of the perturbed parameters. Our study agrees with that of Idrisi^23^ for $e=a=A={\sigma }_{1}={\sigma }_{2}={M}_{b}=0$, Idrisi and Ullah^25,26^ for $A={\sigma }_{1}={\sigma }_{2}={M}_{b}=0, a=1$, Duggad et al.^14^ for $\alpha =\beta ={M}_{b}=0, a=1$.

**Table 5:** Effects of various perturbations on the Critical Mass Value $\left({\mu }_{c}\right)$ of the triangular libration points. $\left(\mu =0.01215, A=0.0000003686, e=0.0549, \gamma =-0.0004527, T=0.01\right)$.

Case	$\alpha$	${\sigma }_{1}$	${\sigma }_{2}$	$k$	${M}_{b}$	${L}_{4}\left(\xi \right)$	${L}_{5}\left(\pm \eta \right)$	${\mu }_{c}$	$Remarks$
1	$0$	$0$	$0$	$0$	$0$	$-0.48785$	$0.864459$	$0.0379644$	Stable
2	$0.5$	$0$	$0$	$0$	$0$	$-0.321183$	$0.768234$	$0.0550862$	Stable
3	$0.5$	$0.03$	$0$	$0$	$0$	$-0.332618$	$0.727205$	$-1.81788$	Unstable
	$0.5$	$0.04$	$0$	$0$	$0$	$-0.336429$	$0.713528$	$-2.44220$	Unstable
	$0.5$	$0.05$	$0$	$0$	$0$	$-0.340241$	$0.699851$	$-3.06652$	Unstable
4	$0.5$	$0.03$	$0.01$	$0$	$0$	$-0.323806$	$0.737994$	$-1.82504$	Unstable
	$0.5$	$0.04$	$0.02$	$0$	$0$	$-0.318806$	$0.7351080$	$-2.45652$	Unstable
	$0.5$	$0.05$	$0.03$	$0$	$0$	$-0.313806$	$0.7322210$	$-3.08800$	Unstable
5	$0.5$	$0.03$	$0.01$	$0.05$	$0$	$-0.996013$	$0.3496070$	$-1.85710$	Unstable
	$0.5$	$0.04$	$0.02$	$0.10$	$0$	$-1.68354$	$-0.0534018$	$-2.66197$	Unstable
	$0.5$	$0.05$	$0.03$	$0.15$	$0$	$-2.39140$	$-0.468146$	$-3.60816$	Unstable
6	$0.050$	$0$	$0$	$0$	$0.2$	$-0.471184$	$0.777405$	$0.0454086$	Stable
	$0.045$	$0$	$0$	$0$	$0.4$	$-0.47285$	$0.700936$	$0.0509693$	Stable
	$0.040$	$0$	$0$	$0$	$0.6$	$-0.474517$	$0.624467$	$0.056530$	Stable
7	$0.050$	$0.03$	$0.01$	$0.05$	$0.2$	$-0.541027$	$0.708327$	$-1.89966$	Unstable
	$0.045$	$0.04$	$0.02$	$0.10$	$0.4$	$-0.593300$	$0.596843$	$-2.55925$	Unstable
	$0.040$	$0.05$	$0.03$	$0.15$	$0.6$	$-0.633348$	$0.492424$	$-3.22706$	Unstable

The critical mass value $\left({\mu }_{c}\right)$ decreases with an increase in the Albedo effects, triaxiality of the smaller primaries, and potential due to belt for the Earth–Moon system.

**Figure 10:**
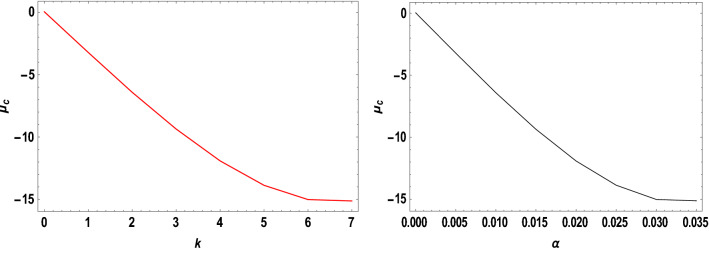
Effects of Albedo parameters on the Critical Mass Value $\left({\mu }_{c}\right)$ of the triangular libration points.

**Figure 11:**
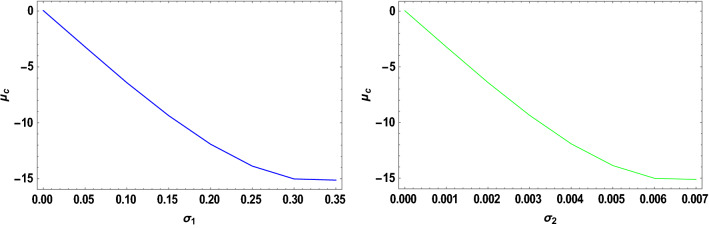
Effects of the triaxiality on the Critical Mass Value $\left({\mu }_{c}\right)$ of the triangular libration points.

**Figure 12:**
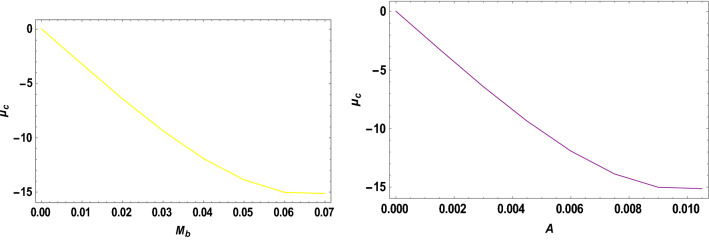
Effects of oblateness and potential due to belt on the Critical Mass Value $\left({\mu }_{c}\right)$ of the triangular libration points.

**Figure 13:**
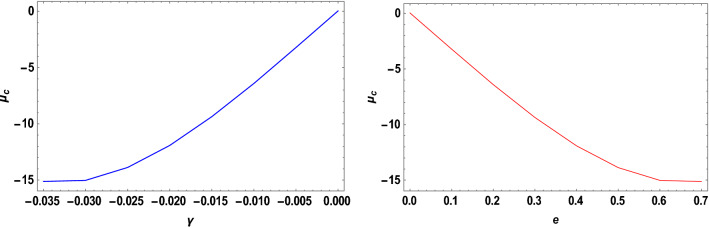
Effects of the semi-major axis and the Eccentricity of the orbits on the Critical Mass Value $\left({\mu }_{c}\right)$ of the triangular libration points.

We have also viewed the region of stability when the problem is considered at varying parameters. As shown in Table 6 and Figs. 10, 11, 12 and 13, the region of stability decreases with an increase in the values of the semi-major axis, Eccentricity of the orbits, Albedo effects, triaxiality, and the potential due to the belt of the problem under study. In the absence of the parameters mentioned above, the triangular libration points are stable (see Table 6). However, with the introduction of the parameters, the triangular libration points are unstable (see Table 6 and Figs. 10, 11, 12 and 13).

**Table 6:** Effects of varying parameters on the Critical Mass Value $\left({\mu }_{c}\right)$ of the triangular libration points. $\left(\mu =0.01215\right)$.

$k$	$\alpha$	${\sigma }_{1}$	${\sigma }_{2}$	${M}_{b}$	$e$	$A$	$\gamma$	$T$	${\mu }_{c}$	Remarks
$0$	$0.00$	$0.00$	$0.00$	$0.00$	$0.00$	$0.0000$	$0.00$	$0.00$	$0.0385209$	Stable
$1$	$0.005$	$0.05$	$0.001$	$0.01$	$0.1$	$0.0015$	$-0.005$	$0.10$	$-3.19006$	Unstable
$2$	$0.010$	$0.10$	$0.002$	$0.02$	$0.2$	$0.0030$	$-0.010$	$0.12$	$-6.32530$	Unstable
$3$	$0.015$	$0.15$	$0.003$	$0.03$	$0.3$	$0.0045$	$-0.015$	$0.14$	$-9.26309$	Unstable
$4$	$0.020$	$0.20$	$0.004$	$0.04$	$0.4$	$0.0060$	$-0.020$	$0.16$	$-11.8826$	Unstable
$5$	$0.025$	$0.25$	$0.005$	$0.05$	$0.5$	$0.0075$	$-0.025$	$0.18$	$-14.0461$	Unstable
$6$	$0.030$	$0.30$	$0.006$	$0.06$	$0.6$	$0.0090$	$-0.030$	$0.20$	$-15.5994$	Unstable
$7$	$0.035$	$0.35$	$0.007$	$0.07$	$0.7$	$0.0105$	$-0.035$	$0.22$	$-16.3711$	Unstable

When the parameters indicated above are increased, the critical mass value $\left({\mu }_{c}\right)$ decreases, reducing the stability region. The unstable behavior of the triangular libration points is depicted in Figs. 10, 11, 12 and 13. The critical mass value for the problem under investigation decreases as the parameters increase.

The figures below demonstrate the effects of the varying parameters, as in Table 6, on the critical mass value $\left({\mu }_{c}\right)$ of the triangular libration points.

We also numerically compute the roots of Eq. (15) to ascertain the stable nature of the triangular libration points. This computation is done with the help of a software package called MATHEMATICA for the Earth–Moon system. The nature of the roots is demonstrated in Table 7.

**Table 7:** Stability of triangular libration points for the Earth–Moon system $\left(\mu =0.01215, A=0.0000003686, e=0.0549, \gamma =-0.0004527, T=0.01\right)$.

Case	$\alpha$	$k$	${\sigma }_{1}$	${\sigma }_{2}$	${M}_{b}$	Stability of triangular libration points		$\mathrm{Remarks}$
						$\pm {\lambda }_{\mathrm{1,2}}$	$\pm {\lambda }_{\mathrm{3,4}}$	
1	$0$	$0$	$0$	$0$	$0$	$-0.720323\pm 0.720323i$	$0.72032\pm 0.720323i$	Unstable
2	$0.5$	$0$	$0$	$0$	$0$	$-0.723404\pm 0.723404i$	$0.723404\pm 0.723404i$	Unstable
3	$0.5$	$0.05$	$0$	$0$	$0$	$-0.766827\pm 0.766827i$	$0.766827\pm 0.766827i$	Unstable
	$0.5$	$0.10$	$0$	$0$	$0$	$-0.779785\pm 0.779785i$	$0.779785\pm 0.779785i$	Unstable
	$0.5$	$0.15$	$0$	$0$	$0$	$-0.792128\pm 0.792128i$	$0.792128\pm 0.792128i$	Unstable
4	$0.5$	$0.05$	$0.03$	$0$	$0$	$-0.754309\pm 0.754309i$	$0.754309\pm 0.754309i$	Unstable
	$0.5$	$0.10$	$0.04$	$0$	$0$	$-0.755436\pm 0.755436i$	$0.755436\pm 0.755436i$	Unstable
	$0.5$	$0.15$	$0.05$	$0$	$0$	$-0.756559\pm 0.756559i$	$0.756559\pm 0.756559i$	Unstable
5	$0.5$	$0.05$	$0.03$	$0.01$	$0$	$-0.758993\pm 0.758993i$	$0.758993\pm 0.758993i$	Unstable
	$0.5$	$0.10$	$0.04$	$0.02$	$0$	$-0.754227\pm 0.754227i$	$0.754227\pm 0.754227i$	Unstable
	$0.5$	$0.15$	$0.05$	$0.03$	$0$	$-0.738241\pm 0.738241i$	$0.738241\pm 0.738241i$	Unstable
6	$0.050$	$0$	$0$	$0$	$0.2$	$-0.719126\pm 0.719126i$	$0.719126\pm 0.719126i$	Unstable
	$0.045$	$0$	$0$	$0$	$0.4$	$-0.717579\pm 0.717579i$	$0.717579\pm 0.717579i$	Unstable
	$0.040$	$0$	$0$	$0$	$0.6$	$-0.716021\pm 0.716021i$	$0.716021\pm 0.716021i$	Unstable
7	$0.050$	$0.05$	$0.03$	$0.01$	$0.2$	$-0.723505\pm 0.723505i$	$0.723505\pm 0.723505i$	Unstable
	$0.045$	$0.10$	$0.04$	$0.02$	$0.4$	$-0.721263\pm 0.721263i$	$0.721263\pm 0.721263i$	Unstable
	$0.040$	$0.15$	$0.05$	$0.03$	$0.6$	$-0.718126\pm 0.718126i$	$0.718126\pm 0.718126i$	Unstable

We discover that for each set of values, there is at least one complex root with a positive real part, as shown in Table 7. As a result, the Earth–Moon system's triangular libration points are unstable in the Lyapunov sense.

## Poincare surfaces of section (PSS)

Stable periodic and quasi-periodic orbits around the primaries are determined with the help of the Poincare Surface of Section (PSS). To determine the PSS of a given Jacobian integral $C+\left({\dot{\xi }}^{2}+{\dot{\eta }}^{2}\right)=2\Omega$, where $C$ is the Jacobi constant, we express $\dot{\eta }$ in terms of the other three variables and then substituting them in the equations of motion for each occurrence of $\dot{\eta },$ we reduce the phase space to a $3-D$ $\left(\xi , \eta ,\dot{\xi }\right)$ the subspace of the original $4-D$. We repeat the action each time the orbit passes through the surface of the section plane with positive $\dot{\eta }$ to determine the remaining variables $\left(\xi , \dot{\xi }\right)$.

As good analytical tools, many kinds of research are carried out using PSS. In the Circular Restricted Three-Body Problem, Singh and Leke^7^ employed PSS to establish the stability of the motion of a passively gravitating dust grain particle in the gravitational field of two big stars. Abduljabar et al.^34^ used oblateness and solar radiation in their work, drawing PSS in the presence and absence of oblateness. Their results reveal that oblateness has little influence, but the PSS shrinks in the presence and lack of solar radiation pressure. Poincare Surface of Section (PSS) using different values of the variation constant $\left({\lambda }_{1}=0.2, 0.6, 1\right)$ were observed by Ansari et al.^35^, their results confirmed that when the value of ${\lambda }_{1}$ increases, the surfaces PSS are shrinking for $\xi \epsilon \left(0.05, 0.17\right)$. In the circular restricted problem of three bodies, Singh and Tyokyaa^33^ used PSS to show the sensitivity to changes in the positions and velocities of triangular libration points.

In our present study, we assume $\xi ={x}_{1}, \eta ={x}_{2}, \dot{\xi }={x}_{3}, \dot{ \eta }={x}_{4}$ and we then reduce the two-second order differential equations of motion given in Eq. (1) to the first-order differential equations as in Eq. (21).

21 $$\begin{aligned} & \frac{{dx_{1} }}{{dt}} = x_{3} \left( t \right), \\ & \frac{{dx_{2} }}{{dt}} = x_{4} \left( t \right), \\ & \frac{{dx_{3} }}{{dt}} = 2x_{4} \left( t \right) + \left( {1 - e^{2} } \right)^{{\frac{{ - 1}}{2}}} \left[ {x_{1} \left( t \right) - \frac{1}{{n^{2} }}\left( {\frac{{\left( {1 - \mu } \right)\left( {1 - \alpha } \right)\left( {x_{1} \left( t \right) - \mu } \right)}}{{r_{1} ^{3} }} + \frac{{3\left( {1 - \mu } \right)\left( {1 - \alpha } \right)\left( {x_{1} \left( t \right) - \mu } \right)A}}{{2r_{1} ^{5} }} + \frac{{\mu \left( {1 - \beta } \right)\left( {x_{1} \left( t \right) + 1 - \mu } \right)}}{{r_{2} ^{3} }} + \frac{{3\mu \left( {2\sigma _{1} - \sigma _{2} } \right)\left( {1 - \beta } \right)\left( {x_{1} \left( t \right) + 1 - \mu } \right)}}{{2r_{2} ^{5} }} - \frac{{15\mu \left( {\sigma _{1} - \sigma _{2} } \right)\left( {1 - \beta } \right)\left( {x_{1} \left( t \right) + 1 - \mu } \right)\eta ^{2} }}{{2r_{2} ^{7} }} + \frac{{x_{1} \left( t \right)M_{b} }}{{\left( {r^{2} + T^{2} } \right)^{{\frac{3}{2}}} }}} \right)} \right], \\ & \frac{{dx_{4} }}{{dt}} = - 2x_{3} \left( t \right) + \frac{{x_{2} \left( t \right)}}{{\left( {1 - e^{2} } \right)^{{\frac{1}{2}}} }}\left[ {1 - \frac{1}{{n^{2} }}\left( {\frac{{\left( {1 - \mu } \right)\left( {1 - \alpha } \right)}}{{r_{1} ^{3} }} + \frac{{3\left( {1 - \mu } \right)\left( {1 - \alpha } \right)A}}{{2r_{1} ^{5} }} + \frac{{\mu \left( {1 - \beta } \right)}}{{r_{2} ^{3} }} + \frac{{3\mu \left( {2\sigma _{1} - \sigma _{2} } \right)\left( {1 - \beta } \right)}}{{2r_{2} ^{5} }} + \frac{{3\mu \left( {\sigma _{1} - \sigma _{2} } \right)\left( {1 - \beta } \right)}}{{r_{2} ^{5} }} - \frac{{15\mu \left( {\sigma _{1} - \sigma _{2} } \right)\left( {1 - \beta } \right)\eta ^{2} }}{{2r_{2} ^{7} }} + \frac{{M_{b} }}{{\left( {r^{2} + T^{2} } \right)^{{\frac{3}{2}}} }}} \right)} \right]. \\ \end{aligned}$$

**Figure 14:**
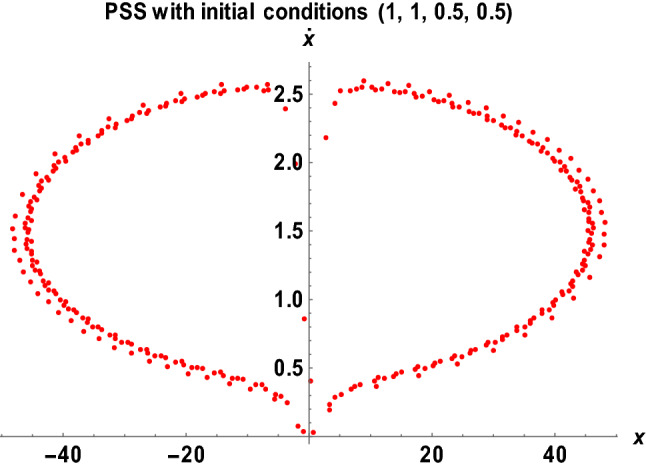
The PSS for the Earth–Moon system with $\mu =0.01215,A=0.0000003686,e=0.0549, a=1.000453, T=0.01, {\sigma }_{1}=0.15, {\sigma }_{2}=0.003,\alpha =0.015, k=3, {M}_{b}=0.03$.

**Figure 15:**
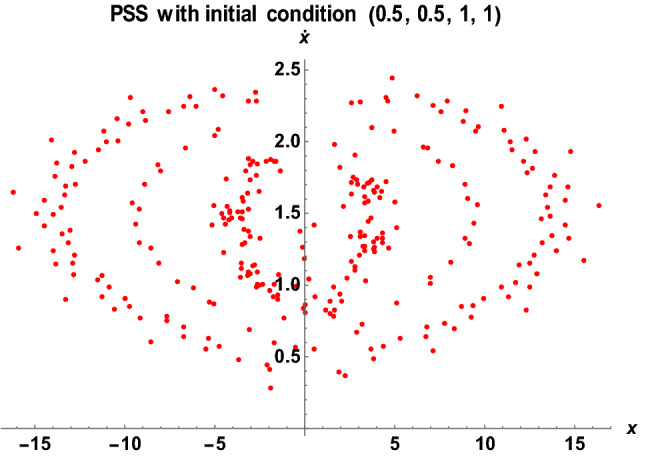
The PSS for the Earth–Moon system with $\mu =0.01215,A=0.0000003686,e=0.0549, a=1.000453, T=0.01, {\sigma }_{1}=0.25, {\sigma }_{2}=0.005,\alpha =0.025, k=5, {M}_{b}=0.05$.

**Figure 16:**
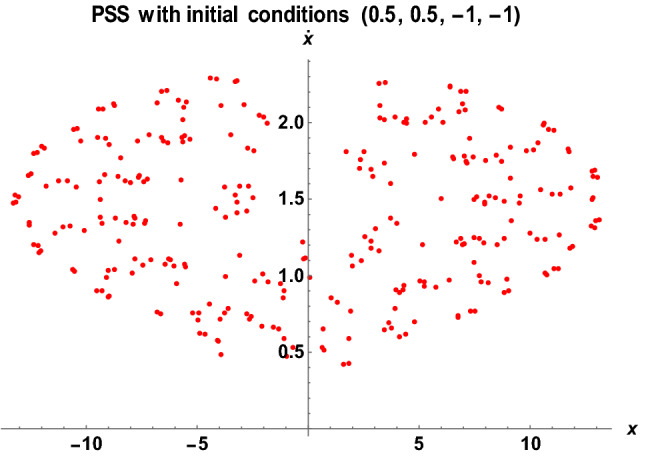
The PSS for the Earth–Moon system with $\mu =0.01215,A=0.0000003686,e=0.0549, a=1.000453, T=0.01, {\sigma }_{1}=0.30, {\sigma }_{2}=0.006,\alpha =0.06, k=6, {M}_{b}=0.06$.

**Figure 17:**
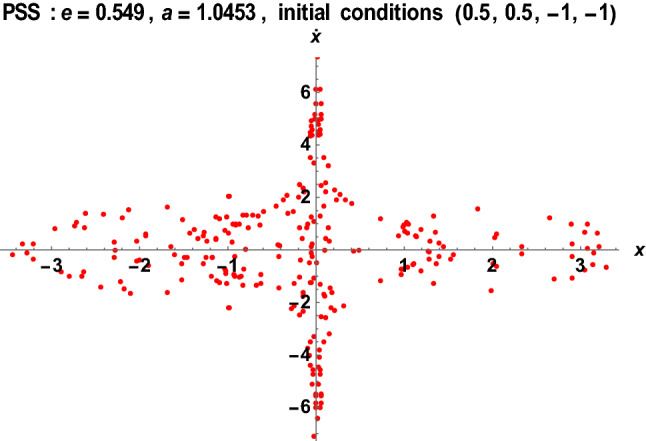
The PSS for the Earth–Moon system with $\mu =0.01215,A=0.0000003686,e=0.549, a=1.0453, T=0.01, {\sigma }_{1}=0.30, {\sigma }_{2}=0.006,\alpha =0.06, k=6, {M}_{b}=0.06$.

Figures 14, 15, 16 and 17 show the stability behavior of periodic and quasi-periodic orbits around the primaries for the Earth–Moon system using the software MATHEMATICA.

The stability behavior of periodic and quasi-periodic orbits around the primary for the Earth–Moon system has been graphically depicted using the Poincare Surface of Section (PSS) (see Figs. 14, 15, 16 and 17). As represented in Fig. 14, when we have considered varying parameters $\left({\sigma }_{1}=0.15, {\sigma }_{2}=0.003,\alpha =0.015, k=3, {M}_{b}=0.03\right)$ with initial conditions (1, 1, 0.5, 0.5), the system possessed two closed-packed layers of onion shape-like orbits with a concentrated number of points in the second layer. We have observed a similar behavior of the orbits in Fig. 15 when we have considered the initial conditions (0.5, 0.5, 1, 1) with varying values of parameters $\left({\sigma }_{1}=0.25, {\sigma }_{2}=0.005,\alpha =0.025, k=5, {M}_{b}=0.05\right)$. As the initial conditions changed, the system formed three spaced layers of onion shape-like orbits, with the inner one having a thick meshed point-like shape (see Fig. 15).

As shown in Fig. 16, the system exhibits irregular merged-points onion shape when the initial conditions (0.5, 0.5, 1, 1) are changed to (0.5, 0.5, − 1, − 1) with the same values of parameters as in Fig. 15. Then, the three layers of onion shape like a Different scenario are observed in Fig. 17 when the semi-major axis and Eccentricity of the orbits are altered with the same initial conditions as in Fig. 16. As demonstrated in Fig. 17, the system possessed irregular star-like shape orbits around the primaries.

Generally, we have observed that the system's behavior has changed significantly with a bit of change in the initial conditions, the semi-major axis, and the Eccentricity of the orbits. We witnessed a chaotic dynamical behavior of the system resulting in either regular or irregular orbits.

## Conclusions

Our study investigates the effects of Albedo in the elliptic restricted three-body problem under an oblate primary, a triaxial secondary, and a potential due to belt for the Earth–Moon system. Analytically, we have described the locations of triangular points and their stability in Eqs. 1- 4, 6, 9, 10, 15, 20, and 21. The numerical data of Table 1 shows that there are no black bodies in our solar systems as all the Albedo values of the planets are greater than zero. The numerical data of Tables 2 and 3 are used to demonstrate the zero velocity curves (ZVC) for the problem under study. As observed in Figs. 3 and 4, when the Albedo effects of both primaries, triaxiality of the smaller primary, and the potential due to the belt of the system increase with an increase in the Jacobian constant $C$, the two smaller obits merged to form a smaller region in between the two major regions resulting to three isolated possible regions.

The movements of the primary regions, as observed in Fig. 5 for the system under review, depend on the values of the Jacobian constant $C$ and the parameters involved. This is because the possible boundary regions of the primary get closer to each other as the Jacobian constant value increases with the perturbed parameters, allowing particles to travel freely from one zone to another and possibly merge the permissible regions.

We have observed from our study that the formation of these equilibrium points depends on the system parameters (Mass ratio $\left(\mu \right)$, Eccentricity $\left(e\right)$, Semi-major axis $\left(a\right)$, Oblateness $\left(A\right)$, Albedo (*α* and *β*), Triaxiality (*α*_1_ and *α*_2_) and the Potential due to belt $\left({M}_{b}\right)$) of the problem under study. Our study shows that as the parameters increase, the position ${L}_{4}$ and ${L}_{5}$ move towards the origin for cases 1, 2, 3, and 4 and away from it for cases 5, 6, and 7 for the Earth–Moon system (see Table 4 and Fig. 9).

Considering the range $0<\mu <{\mu }_{c}$ for stable libration points and ${\mu }_{c}\leq\mu \le \frac{1}{2}$ for unstable libration points, our study has established that the triangular libration points are stable and unstable for cases 1, 2, and 6 and cases 3, 4, 5, and 7, respectively (see Table 5). As presented in Table 7, there is at least one complex root with a positive real part for each set of values. Hence, the triangular libration points for the Earth–Moon system are unstable in the sense of Lyapunov.

Generally, we have observed that the system's behavior has changed significantly with a bit of change in the initial conditions, the semi-major axis, and the Eccentricity of the orbits. In addition, it is also observed that a chaotic dynamical behavior of the system results in either regular or irregular orbits.

## Data Availability

All data generated or analyzed during the current study are included in this manuscript.
